# Nitrogenated holey two-dimensional structures

**DOI:** 10.1038/ncomms7486

**Published:** 2015-03-06

**Authors:** Javeed Mahmood, Eun Kwang Lee, Minbok Jung, Dongbin Shin, In-Yup Jeon, Sun-Min Jung, Hyun-Jung Choi, Jeong-Min Seo, Seo-Yoon Bae, So-Dam Sohn, Noejung Park, Joon Hak Oh, Hyung-Joon Shin, Jong-Beom Baek

**Affiliations:** 1 School of Energy and Chemical Engineering/Low-Dimensional Carbon Materials Center, Ulsan National Institute of Science and Technology (UNIST), 100 Banyeon, Ulsan 689-798, South Korea; 2 Department of Chemical Engineering, Pohang University of Science and Technology (POSTECH), 77 Cheongam, Pohang 790-784, South Korea; 3 School of Materials Science and Engineering/Low-Dimensional Carbon Materials Center/KIST-UNIST Ulsan Center for Convergent Materials, Ulsan National Institute of Science and Technology (UNIST), 100 Banyeon, Ulsan 689-798, South Korea; 4 Department of Physics, Ulsan National Institute of Science and Technology (UNIST), 100 Banyeon, Ulsan 689-798, South Korea

## Abstract

Recent graphene research has triggered enormous interest in new two-dimensional ordered crystals constructed by the inclusion of elements other than carbon for bandgap opening. The design of new multifunctional two-dimensional materials with proper bandgap has become an important challenge. Here we report a layered two-dimensional network structure that possesses evenly distributed holes and nitrogen atoms and a C_2_N stoichiometry in its basal plane. The two-dimensional structure can be efficiently synthesized via a simple wet-chemical reaction and confirmed with various characterization techniques, including scanning tunnelling microscopy. Furthermore, a field-effect transistor device fabricated using the material exhibits an on/off ratio of 10^7^, with calculated and experimental bandgaps of approximately 1.70 and 1.96 eV, respectively. In view of the simplicity of the production method and the advantages of the solution processability, the C_2_N-*h*2D crystal has potential for use in practical applications.

## Abstract (web summary)

There is currently interest in two-dimensional graphene-like materials incorporating heteroatoms. Here, the authors synthesize a solution-processable, holey two-dimensional network with C_2_N stoichiometry containing evenly distributed holes and nitrogen atoms, and use it to fabricate a field effect transistor.

## Graphical abstract

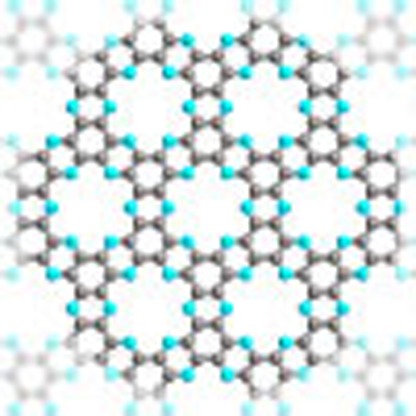

The recent discovery of graphene has garnered interest from researchers in various fields, primarily because of its peculiar inherent monoatomic two-dimensional (2D) crystal electronic structure^1^. The properties of graphene promise many applications such as nanoelectronics^2^, hydrogen storage^3^, batteries^4^ and sensors^5^. The plentiful scientific discussions in the field of graphene research have triggered huge interest in new 2D ordered crystals constructed by inclusion of elements other than carbon^6^. Scientists around the world are working towards the synthesis of 2D crystals with tuneable structures and properties using a bottom-up approach^7^,^8^,^9^,^10^. One of the strongest motivations is the possibility of establishing a finite dimension stable bandgap^9^ in a well-defined 2D structure^10^, which is one of the fundamental prerequisites for a material to be used as an active switching element in electronics^11^,^12^. Various structural modifications, including doping of heteroatoms, have been tested with this goal in mind. Among these modifications, the substitution of nitrogen (N) atoms appears to be an excellent choice because its atomic size and five-electron valence structure (*sp*^2^ hybridization) allow it to naturally fit into a strong covalent network structure of carbon atoms^13^. The multiformity of the technological applications of bandgap-created 2D materials can aid in the search for easy and simple routes to produce N-containing 2D structures. Recently, a variety of techniques have been used to obtain N-containing 2D crystals from graphene^14^,^15^ and graphene oxide^16^; however, these methods offer poor control, involve toxic reagents and harsh reaction conditions and tend to result in metal contamination. Most importantly, however, the structures of N-containing 2D materials are not well-defined for practical use. As a result, creating a properly controlled large-scale production protocol for an N-containing 2D framework has become a substantial challenge for the scientific community^7^,^8^,^17^.

Here, we design and prepare a 2D crystal with uniform holes and nitrogen atoms. The structure and bandgap of the prepared 2D crystal is studied using experimental techniques and density functional theory (DFT). Furthermore, the prepared N-containing holey 2D crystal may be a foundational material for the future development of multifunctional 2D crystals.

## Results

### Synthesis and characterization of C_2_N-*h*2D crystals

**Figure 1:**
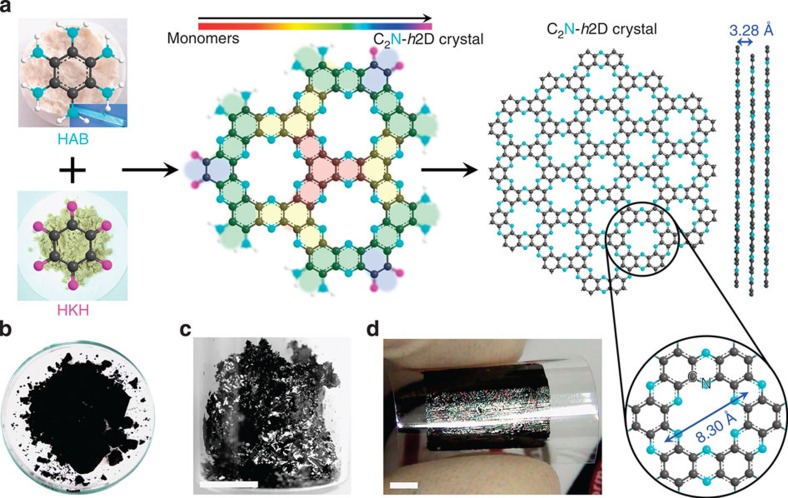
Preparation and structure. (**a**) Schematic representation of the reaction between hexaaminobenzene (HAB) trihydrochloride and hexaketocyclohexane (HKH) octahydrate to produce the C_2_N-*h*2D crystal. The inset in the image of HAB is a polarized optical microscopy image of the HAB single crystal. Digital photographs: (**b**) as-prepared C_2_N-*h*2D crystal; (**c**) solution-cast C_2_N-*h*2D crystal on a SiO_2_ surface after heat-treatment at 700 °C; (**d**) a C_2_N-*h*2D crystal film (thickness: approximately 330 nm) transferred onto a PET substrate. The shiny metallic reflection of the sample indicates that it is highly crystalline.

The difference between graphene (Supplementary Fig. 1a) and holey graphene (Supplementary Fig. 1b) is that the latter has uniform periodic holes in a fused aromatic network structure. However, the holey graphene has not yet been previously developed and the chance is very low. The structure of the newly synthesized holey nitrogenated 2D crystal (Supplementary Fig. 1c) not only has uniform holes, but the holes and phenyl rings are also surrounded by aromatic nitrogen atoms (cyan blue spheres in Supplementary Fig. 1c). In contrast to the fully conjugated π-electron structures of graphene (Supplementary Fig. 1a), an ordered inclusion of uniform holes and nitrogen atoms is expected to widen the gap between the valence and conduction bands (that is, the bandgap) to a level ideal for a bandgap-opened material, which would be useful, for example, in semiconductor applications. The unique N-containing holey 2D crystal was simply synthesized by the reaction between hexaaminobenzene (HAB) trihydrochloride (Fig. 1a)^18^ and hexaketocyclohexane (HKH) octahydrate in *N*-methyl-2-pyrrolidone (NMP) in the presence of a few drops of sulphuric acid (H_2_SO_4_) or in trifluoromethanesulphonic acid. The tremendous potential energy gain by aromatization (approximately −89.7 kcal Umol^−1^, calculated using DFT, Supplementary Fig. 2) is responsible for the spontaneous polycondensation between the HAB and HKH and leads to the formation of a layered crystalline 2D network structure (Supplementary Fig. 2)^19^. The resultant dark-black graphite-like solid (Fig. 1b), whose appearance was a strong indication of the formation of a conjugated layered 2D crystal, was Soxhlet extracted with water and then methanol, respectively, to completely remove any small mass impurities and was finally freeze-dried at −120 °C under reduced pressure (0.05 mm Hg). Utilizing such a strong driving force for the aromatization, the 3D fused π-conjugated microporous polymers were also conveniently realized by solvothermal reaction in the sealed glass tube^20^ and ionothermal process in the presence of AlCl_3_ for energy storage^21^. When the sample solution was cast onto a SiO_2_ substrate, annealed at 700 °C under an argon atmosphere and collected by etching in hydrofluoric acid, the solution contained shiny flakes observable under a strong light (Fig. 1c). A large-area film was also cast and transferred onto a flexible polyethylene terephthalate (PET) substrate (Fig. 1d).

The empirical formulas of the product are C_2_N for the repeating unit in the basal plane (structure **2** in Supplementary Fig. 2) and C_6_H_2_N_3_O for the entire molecule, including the edge functional the groups (structure **1** in Supplementary Fig. 2). Various elemental analyses using different techniques confirmed the chemical formula of the molecule (Supplementary Table 1). Hence, we named the 2D crystal ‘C_2_N holey 2D crystal’ or ‘C_2_N-*h*2D crystal’ and determined it to be soluble in various commonly used solvents, in which it exhibits colloidal scattering (Supplementary Fig. 3).

The powder X-ray diffraction (XRD) pattern of the C_2_N-*h*2D crystal indicates that its structure is readily layered and highly crystalline. Like the XRD pattern of graphite, the pattern of this crystal also shows a sharp 002 diffraction peak at 27.12° (Supplementary Fig. 4a), whose position corresponds to an interlayer distance (*d*-spacing) of 0.328 nm. However, this *d*-spacing is narrower than the *d*-spacing of graphite (*d*=0.335 nm)^22^. The narrower *d*-spacing of the C_2_N-*h*2D crystal is thought to originate from the evenly distributed nitrogen atoms and holes. Nitrogen has a smaller atomic size (70 pm) and greater electronegativity (*χ*=3.07) than carbon (77 pm and *χ*=2.55). In addition to van der Waals forces, the C_2_N-*h*2D crystal exhibits polar attraction, resulting in stronger interlayer interactions than those in graphite.

X-ray photoelectron spectroscopy (XPS) measurements were performed to probe the chemical composition of the new material. The characteristic band for the K-edge of nitrogen appeared at 399 eV, indicating the presence of *sp*^*2*^-hybridized nitrogen atoms in the holey 2D structure. The survey scan spectrum from the XPS analysis revealed the presence of C1s, N1s and O1s without any other impurities (Supplementary Fig. 4b, Supplementary Note 1). The corresponding high-resolution XPS spectra and the XPS spectra of the heat-treated samples are presented in Supplementary Figs 5 and 6, respectively. Thermogravimetric analysis indicated that the as-prepared C_2_N-*h*2D crystal underwent a gradual weight loss from the beginning of the scan (Supplementary Fig. 4c). However, the C_2_N-*h*2D crystal heated at 700 °C under an argon atmosphere exhibited high thermal stability under both air and argon (Supplementary Fig. 4d), indicating that the early weight loss of the as-prepared sample was due to the volatilization of entrapped substances in the holes. As a result, the Brunauer–Emmett–Teller-specific surface areas of the untreated and heat-treated samples were 26 and 281 m^2^ g^−1^, respectively.

**Figure 2:**
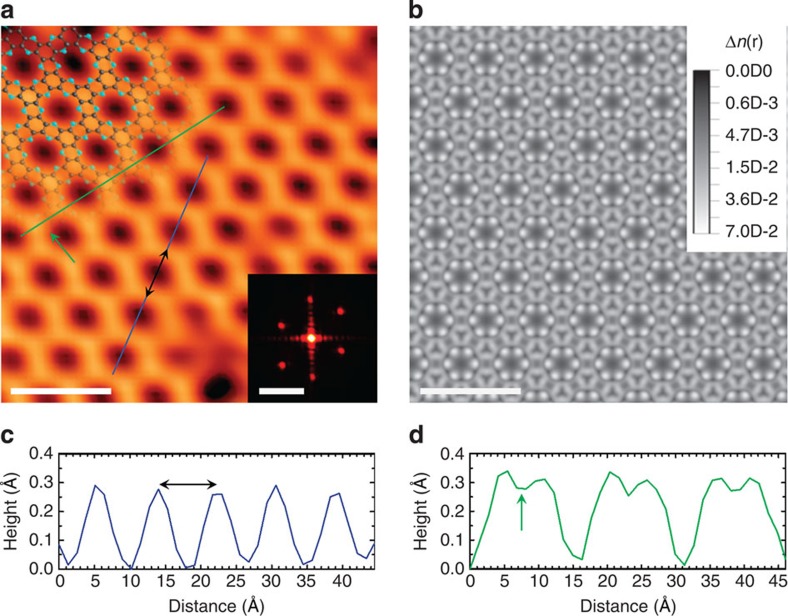
STM characterization. (**a**) An atomic-resolution STM topography image of the C_2_N-*h*2D crystal on Cu(111). The STM image was obtained at a sample bias of 0.7 V and a tunnelling current of 300 pA. The top-left inset is the structure of the C_2_N-*h*2D crystal superimposed on the image. The bottom-right inset is 2D fast Fourier transform. (**b**) Simulated image (see the first-principles calculations in ESI). (**c**) The topographic height profile along deep-blue line. (**d**) The topographic height profile along green line. Green arrow indicates the location of the C–N bridged region. The scale bars in (**a**), the inset in (**a**) and (**b**) are 2.0 nm, 2.0 nm^−1^ and 2.0 nm, respectively.

The bulk morphologies of the 2D crystals were examined using field-emission scanning electron microscopy. The grain sizes of the as-prepared and heat-treated samples were as large as a few hundred micrometres (Supplementary Fig. 7a–c). The transmission electron microscopy (TEM) image obtained from the dispersed sample appears to show a wrinkled morphology (Supplementary Fig. 7d), which is attributed to the flexible nature of the holey 2D structure^23^. The high-magnification TEM image indicates high crystallinity (Supplementary Fig. 7e) with an interlayer *d*-spacing of 0.327 nm (Supplementary Fig. 7f), which is in good agreement with the XRD results (0.328 nm, see Supplementary Fig. 4a), confirming that the C_2_N-*h*2D crystal has the thinnest layered 2D structure reported to date. TEM element maps obtained by energy-dispersive X-ray spectroscopy and electron energy loss spectroscopy indicate that the elemental compositions of the samples are in accordance with the theoretical values (Supplementary Fig. 8). Uniform films with various thicknesses were also cast onto a SiO_2_(300 nm)/Si wafer (Fig. 1d and Supplementary Fig. 9). We performed scanning tunnelling microscopy (STM) experiments to verify the molecular structure of the C_2_N-*h*2D crystal. A single-layer C_2_N-*h*2D crystal sample was deposited thermally onto a Cu(111) substrate under ultrahigh vacuum (UHV) conditions (Supplementary Fig. 10). Figure 2a shows a high-resolution STM image of the C_2_N-*h*2D monolayer on the Cu(111) substrate. The STM image clearly reveals the uniformly distributed holey structure in hexagonal arrays (left inset, Fig. 2a), which matches precisely with the theoretically derived image (Fig. 2b). The inter-hole distance measured from the height profiles and the 2D fast Fourier transform image is approximately 8.24±0.96 Å (Fig. 2c). The topographic height difference between the holes and the hexagonal lattice is 0.27±0.017 Å, and the benzene rings are imaged slightly higher than the C–N bridged regions (Fig. 2d), contributing to the narrower interlayer *d*-spacing than in graphite (Supplementary Fig. 11a) and *h*-BN (Supplementary Fig. 11b).

**Figure 3:**
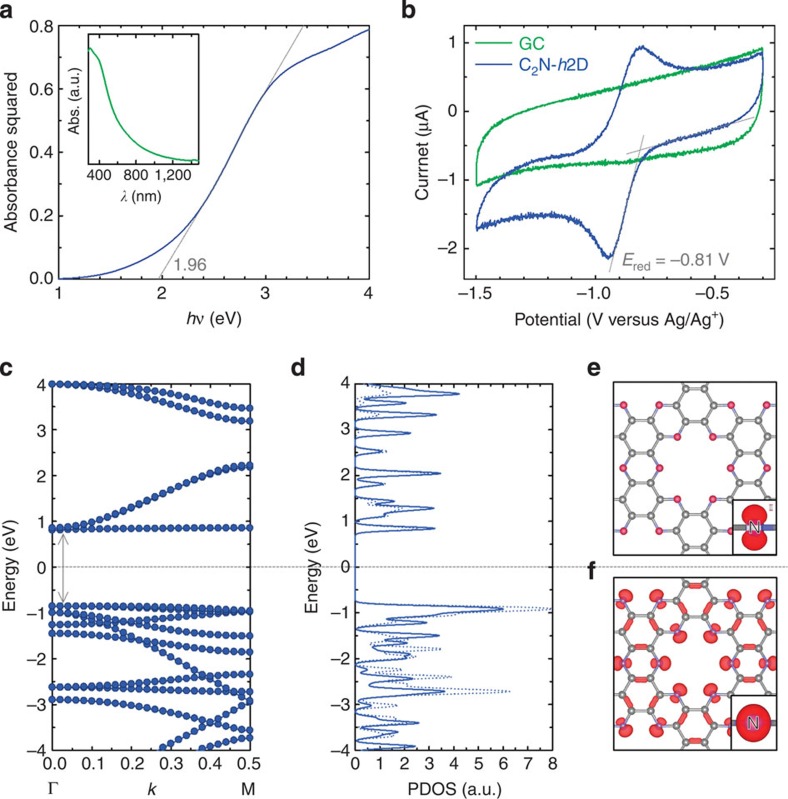
Experimental and theoretical band gap calculations. (**a**) Results of optical band-gap measurements and a plot of the absorbance squared vs. photon energy (*hν*) extrapolated to zero absorption. The inset is the ultraviolet absorption curve. (**b**) Cyclic voltammograms of the C_2_N-*h*2D crystal at a scan rate of 100 mV s^−1^ using a Ag/Ag^+^ reference electrode. (**c**) The band structure from the zone centre to the M point of the 2D triangular lattice. (**d**) The density of electronic states. An iso-surface plot of the Kohn–Sham orbital at the gamma point: (**e**) the conduction-band minimum state; (**f**) the doubly degenerate valence-band maximum state. The insets in **e** and **f** signify the *p* and *σ*-orbital characters of the corresponding bands.

The extensively investigated 2D crystals of graphene (Supplementary Fig. 11a,d) and *h*-BN (Supplementary Fig. 11b,e) are fundamentally different in terms of their electronic structures, despite their geometrical similarity. For example, graphene is a conductor with a vanishingly small bandgap^24^, whereas *h*-BN is an insulator with a wide bandgap of 5.05–6.40 eV (ref. 24). Thus, the electronic structure of the newly developed C_2_N-*h*2D crystal is worthy of investigation. The direct bandgap was empirically determined using ultraviolet–visible spectroscopy (Fig. 3a): it is approximately 1.96 eV, which is well within the range of semiconductor bandgaps^25^. To elucidate the band structure of the C_2_N-*h*2D crystal, we measured cyclic voltammograms to determine the onset reduction potential, which corresponds to the bottom of the conduction band, or the lowest unoccupied molecular orbital (LUMO). To acquire the cyclic voltammograms (Fig. 3b), we deposited a C_2_N-*h*2D crystal onto glassy carbon as a working electrode. Relative to an Ag/Ag^+^ reference electrode, the onset reduction potential appeared at −0.81 V. The LUMO was calculated from the reduction potential to be −3.63 eV (Supplementary Fig. 12). On the basis of the direct optical bandgap of the C_2_N-*h*2D crystal (Fig. 3a), the top of the valence band, or highest occupied molecular orbital, was calculated to be −5.59 eV (Supplementary Note 2).

### Theoretical calculations

We also conducted first-principles DFT calculations to investigate the electronic structure of the C_2_N-*h*2D crystal (Supplementary Methods). The band structure along the symmetry line in the Brillouin zone, from Γ to M, and the density of electronic states are shown in Fig. 3c,d, respectively. According to the gradient-corrected DFT calculations, the C_2_N-*h*2D crystal shows a finite band-gap of approximately 1.70 eV (Fig. 3c), which is smaller (by approximately 0.26 eV) than the optically determined value (1.96 eV). The underestimation of the Kohn–Sham treatment of the DFT is well known^26^. The magnitude of the bandgap and the existence of flat bands near the Fermi levels suggest that the C_2_N-*h*2D crystal is a completely different 2D material from graphene and *h*-BN. In the C_2_N-*h*2D crystal, the benzene rings are bridged by pyrazine rings, which consist of a six-membered D2h ring with two nitrogen atoms facing each other (Fig. 1a and Supplementary Fig. 2). As such, the π-electronic structure of the benzene ring is isolated, resulting in unusual flat bands (whereas graphene has cone-shaped bands) near the edges of the valence and conduction bands. The conduction band minimum consists of a flat band that originates from the localized *p* orbital of the nitrogen atoms (Fig. 3e) and one dispersive band delocalized over the entire plane. The valence-band maximum consists of doubly degenerate flat bands, which originate predominantly from the non-bonding *σ*-states localized at the nitrogen atoms (Fig. 3f). The flat bands near the band edges can be engineered to produce useful phenomena. For example, hole-doping could result in a magnetic state whose spins originate from the two flat bands. Therefore, this material can offer complementary features to the more widely studied graphene, which has a vanishing bandgap (that is a conductor) and *h*-BN, which has a wide bandgap (that is an insulator).

### FET device properties of the C_2_N-*h*2D crystal

**Figure 4:**
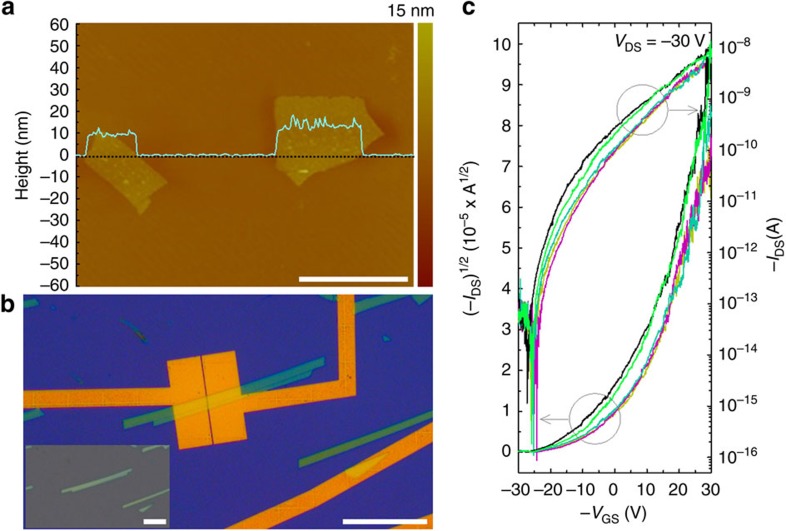
Field-effect transistor (FET) device study. (**a**) Atomic force microscopy image of the C_2_N-*h*2D crystal; the scale bar is 7 μm. The height profile (cyan-blue line) was obtained along the cyan-blue line. (**b**) Optical microscopy image of a C_2_N-*h*2D crystal FET prepared on a SiO_2_(300 nm)/n^++^ Si wafer. The channel length (*L*) of the device is 500 nm, and the channel width-to-length (*W*/*L*)=13. The inset is an optical microscopy image taken before the deposition of Au electrodes on the crystal. The scale bars are 60 μm. (**c**) Transfer curves of the C_2_N-*h*2D crystal FET devices measured at 25 °C under 5 × 10^−6^ torr (*V*_DS_=−30 V).

To illustrate the electrical properties, field-effect transistors (FETs) were fabricated using C_2_N-*h*2D crystals as the active layer. A schematic of the details of film preparation by solution casting and device fabrication are presented in Supplementary Fig. 13, see also Supplementary Note 3. The optical images of typical C_2_N-*h*2D crystal flakes are presented in Supplementary Fig. 14. Because of stronger interlayer interactions, the isolation of a single layer was not possible using this method. Atomic force microscopy analysis of the C_2_N-*h*2D crystal flakes revealed that the average (mean) thickness of the sample (out of ten samples) was 8.0±3.5 nm (Fig. 4a), implying that multilayers of the C_2_N-*h*2D crystal were stacked. Figure 4b shows the optical image of the fabricated FET device, and the inset shows the C_2_N-*h*2D crystals before the deposition of gold electrodes. The devices were annealed at 100 °C under reduced pressure (5 × 10^−6^ torr) to remove chemical impurities that might have been trapped and/or adsorbed into holes and interlayers during the fabrication process.

Typical transfer curves of the C_2_N-*h*2D crystal FET devices are presented in Fig. 4c, and the electrical properties of the C_2_N-*h*2D crystals are summarized in Supplementary Table 2. The on/off current ratio of the transistor was defined as the ratio between the maximum and minimum drain currents; the maximum on/off current ratio obtained from 50 FET devices was 4.6 × 10^7^. Furthermore, when the off current was defined as the average drain current before the turn-on state, the average on/off current ratio remained as high as 2.1 × 10^5^ with a standard deviation of±3.9 × 10^5^. These results clearly indicate that the C_2_N-*h*2D crystals possess a bandgap. In addition, the corresponding output characteristics exhibited well-defined field-effect behaviours under hole-enhanced operation (Supplementary Fig. 15a). Because the work function of gold (approximately 5.10 eV) is much closer to the highest occupied molecular orbital level (−5.59 eV) of the C_2_N-*h*2D crystal than to its LUMO level (−3.63 eV), p-type operation is clearly favourable with gold electrodes (see Supplementary Fig. 16 for the energy-level diagram). Interestingly, the C_2_N-*h*2D crystal exhibited semimetallic (graphene-like) behaviour before annealing (Supplementary Fig. 15b), showing ambipolar charge transport with a Dirac point of −7 V, an electron mobility of 13.5 cm^2^ V^−1^ s^−1^ and a hole mobility of 20.6 cm^2^ V^−1^ s^−1^. The semimetallic behaviour is attributed to the unintentional doping effects by the trapped impurities and/or adsorbed gases in the holey C_2_N-*h*2D crystal structure, thereby suggesting that the electronic properties of the C_2_N-*h*2D crystal are tuneable.

## Discussion

To the best of our knowledge, this work represents the synthesis of micrometre-sized 2D holey crystals with high crystallinity via a simple wet-logical reaction as a bottom-up approach without template assistance. The unique geometric and electronic structure of the C_2_N-*h*2D crystals can be further exploited for use in numerous potential applications for which graphene and *h*-BN have inherent limitations. Comparisons between the C_2_N-*h*2D crystal and other related materials are shown in Supplementary Fig. 17, and their characteristics are summarized in Supplementary Table 3. For example, multifunctionality stemming from uniformly distributed holes and nitrogen atoms is highly attractive for many interesting applications (Supplementary Fig. 18). Specifically, purely organic non-metal magnetism could be achieved after engineering a hole-doping level^27^; size and shape selective absorption of transition metals and biomolecules could be induced via coordinative interactions^28^,^29^,^30^; and new catalysts could be developed for the oxygen reduction reaction^15^ and various organic reactions (see Supplementary Fig. 19, Supplementary Methods).

In summary, we have established that the thinnest layered 2D crystal (designated as C_2_N-*h*2D crystal) reported to date can be simply synthesized via a bottom-up wet-chemical reaction. The crystal has evenly distributed holes and nitrogen atoms in the layered structure with high crystallinity; we verified its structure by atomic-resolution STM imaging. The crystal exhibits *sp*^*2*^ hybridization features with a semiconducting bandgap of approximately 1.96 eV (DFT calculated value: 1.70 eV) with unusual flat bands. The FET device exhibits a 10^7^ on/off ratio, confirming the semiconducting nature of the C_2_N-*h*2D crystal. There are many known difficulties involved in the synthesis of graphene and *h*-BN, and the synthesis of the C_2_N-*h*2D crystals is a simple and highly efficient method for the formation of a fused aromatic 2D network structure. This unique material will open new opportunities in materials science and technology and thus broaden the horizon of applications in electronics, sensors, catalysis and many more research areas, which may lead to complementary uses for graphene and *h*-BN. Furthermore, successful synthesis using a simple and powerful conceptual wet-chemistry-based bottom-up approach coupled with the versatility of organic synthesis may open a new chapter in the cost-effective generation of other 2D materials with tuneable properties, which will be a flourishing new area of research.

## Methods

### Synthesis of the C_2_N-*h*2D

HAB (2 g, 7.20 mmol) and HKH (2.248 g, 7.20 mmol) were charged in a three-necked round bottom flask under argon atmosphere and placed in ice bath. Deoxygenated NMP (80 ml) with a few drops of sulfuric acid or freshly distilled trifluoromethanesulfonic acid (80 ml) was slowly added. The reaction flask was allowed to warm up to room temperature for 2 h. The ice bath was replaced with oil bath and heated to 175 °C for 8 h. Then, the flask was cooled to room temperature and water was added. The solid product that precipitated was collected by suction filtration using polytetrafluoroethylene (PTFE) (0.5 μm) membrane. The resultant dark solid was further Soxhlet extracted with methanol and water, respectively, and freeze-dried at −120 °C under reduced pressure (0.05 mm Hg) for 3 days.

### STM experiments

The STM experiments were carried out in a UHV low-temperature scanning tunnelling microscope (SPECS JT-STM) at 77 K. The Cu(111) single crystal was cleaned by a few cycles of Ar^+^ sputtering and annealing. After cleaning the Cu(111) substrate, the solution-synthesized C_2_N-*h*2D crystal was deposited on the pre-cleaned Cu(111) substrate by *in-situ* thermal evaporation under UHV condition. The sample evaporation temperature was about 600 K, and the temperature of the substrate was maintained at room temperature. To simulate the STM image, we integrated the Kohn–Sham charge density in the energy window of 0.7 eV below and above the Fermi level. The shown image in Fig. 2b is the conduction bands part of the charge density in the plane 1 Å about the atomic layer.

### Preparation of thin films by solution casting

Large-area films were fabricated by drop casting of the C_2_N-*h*2D crystal dispersed in trifluoromethanesulfonic acid on the preheated (140 °C) SiO_2_(300 nm)/Si substrate and subsequently heat-treated at 700 °C in argon for 2 h. Before transferring the solution-casted films on the other substrates, poly(methylmethaacrylate) (PMMA) solution was spin coated on the holey structure films. The SiO_2_ substrate was etched off by an aqueous solution of 2% hydrofluoric acid. Then, the PMMA-coated C_2_N-*h*2D crystal films were transferred on PET substrate and the PMMA was washed off by immersing in acetone and dichloromethane to produce C_2_N-*h*2D crystal films on PET (Fig. 1d). Large-area films on various other substrates such as quartz and glass can be readily prepared through similar procedure, showing more or less very similar results.

### Material characterization

Themogravimetric analysis was conducted in air and argon atmospheres at a heating rate of 10 °C min^−1^ using a Thermogravimetric Analyzer Q200 TA Instrument. Scanning electron microscope images were taken on Field Emission Scanning Electron Microscope Nanonova 230 FEI. XPS was performed on X-ray Photoelectron Spectroscopy Thermo Fisher K-alpha. XRD studies were taken on High Power X-Ray Diffractometer D/MAZX 2500V/PC (Cu–Kα radiation, 35 kV, 20 mA, *λ*=1.5418 Å), Rigaku. Conventional TEM was performed by using JEM-2100F (JEOL) under an operating voltage of 200 keV. The samples for TEM were prepared by drop casting NMP dispersion on Quantifoil holey carbon TEM grid and dried in oven at 80 °C.

## Author contributions

J.-B.B. conceived the C_2_N-*h*2D crystal and oversaw all the research phases. J.M. and J.-B.B. designed the experiments and interpreted the data. J.M. conducted the syntheses and characterizations. M.J., S.-D.S. and H.-J.S. conducted the STM studies. E.K.L, S.-Y.B. and J.H.O carried out the FET device study. J.-M.S., H.-J.C. and I.-Y.J conducted the electrochemical study. N.P. and D.S. were involved in the *ab initio* study of the new material by DFT. S.-M.J., J.-M.S. and H.-J.C. were involved in the TEM experiments. J.-B.B., J.M., N.P., J.H.O. and H.-J.S. wrote the paper and discussed the results. All authors contributed to and commented on this manuscript.

## Additional information

**How to cite this article**: Mahmood, J. *et al*. Nitrogenated holey two-dimensional structures. *Nat. Commun*, 6:6486 doi: 10.1038/ncomms7486 (2015).

## Supplementary Material

Supplementary Information Supplementary Figures 1-19, Supplementary Tables 1-3, Supplementary Notes 1-3, Supplementary Methods and Supplementary References
