# Supplementary material for: Nitrogenated holey two-dimensional structures
Source: Nat Commun. 2015 Mar 6;6:6486. doi: 10.1038/ncomms7486 (PMC4366516; doi:10.1038/ncomms7486)
Supplement: Supplementary Information — Supplementary Figures 1-19, Supplementary Tables 1-3, Supplementary Notes 1-3, Supplementary Methods and Supplementary References [file ncomms7486-s1.pdf]

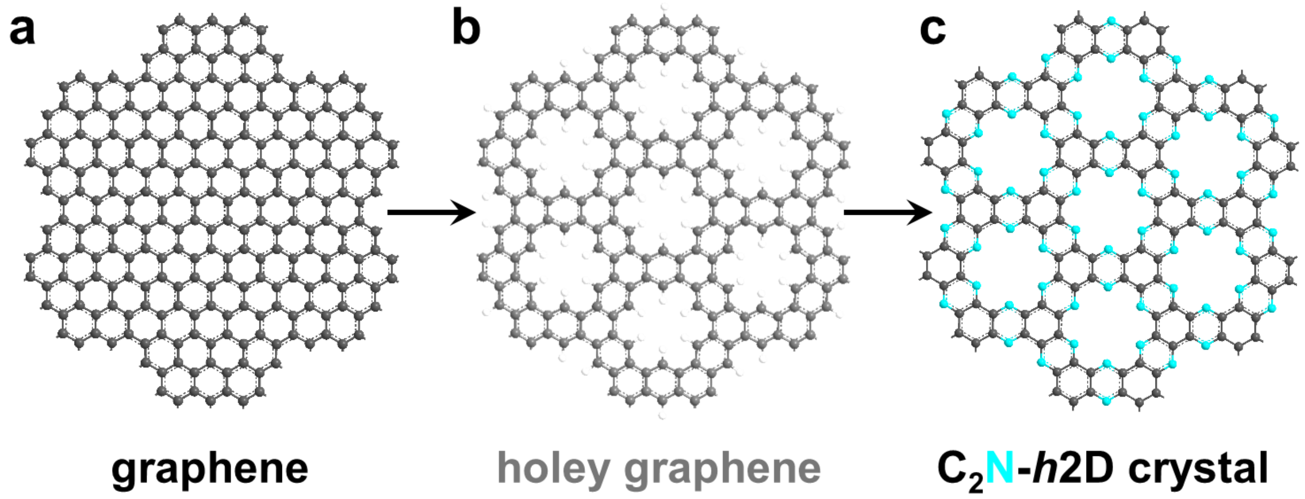

Supplementary Figure 1 | Schematic presentation of the difference among two-dimensional structures. **(a)** Graphene (1st generation). **(b)** Holey graphene (2nd generation, not yet been realized). **(c)** Holey nitrogenated graphene (3rd generation, designated as C_2N-*h*2D crystal).

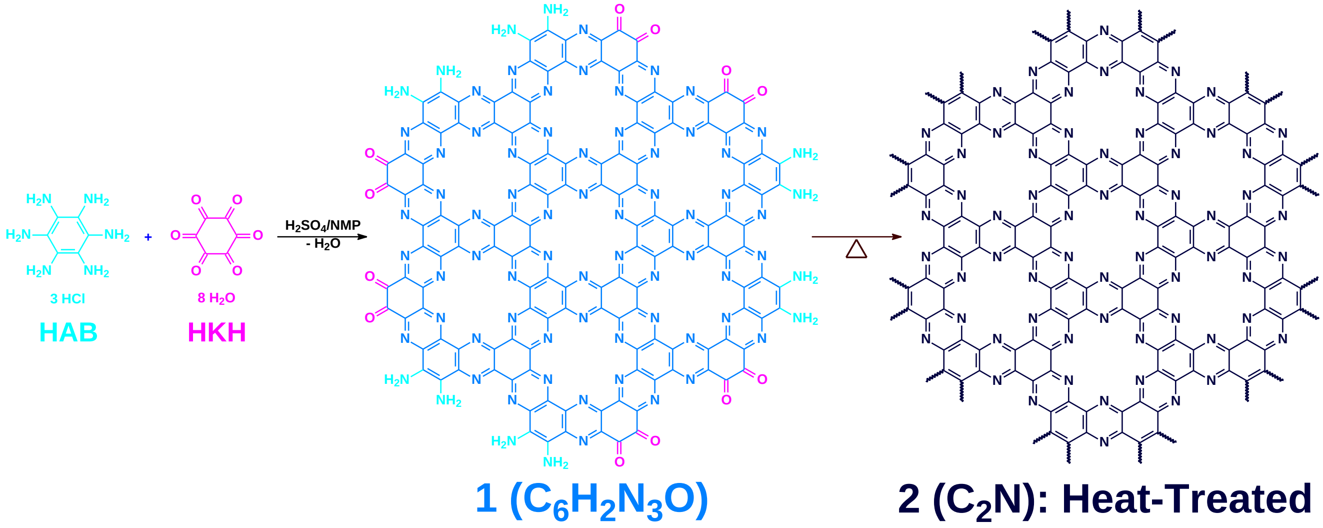

$$C_6H_{12}N_6 + C_6O_6 \longrightarrow 6C_2N + 6H_2O \qquad \Delta H = -89.7 \text{ kcal/mol}$$

Supplementary Figure 2 | Schematic presentation for the formation of the C_2N-*h*2D crystal with edge groups and thermodynamics of reaction. Reaction enthalpy (ΔH), calculated with the PBE density functional theory, is found to be -89.7 kcal/unit mol. After heat-treatment, bound small molecules (H_2O, O_2, and etc.) and edge groups from structure **1** could be thermally stripped off to give the heat-treated C_2N holey structure **2**.

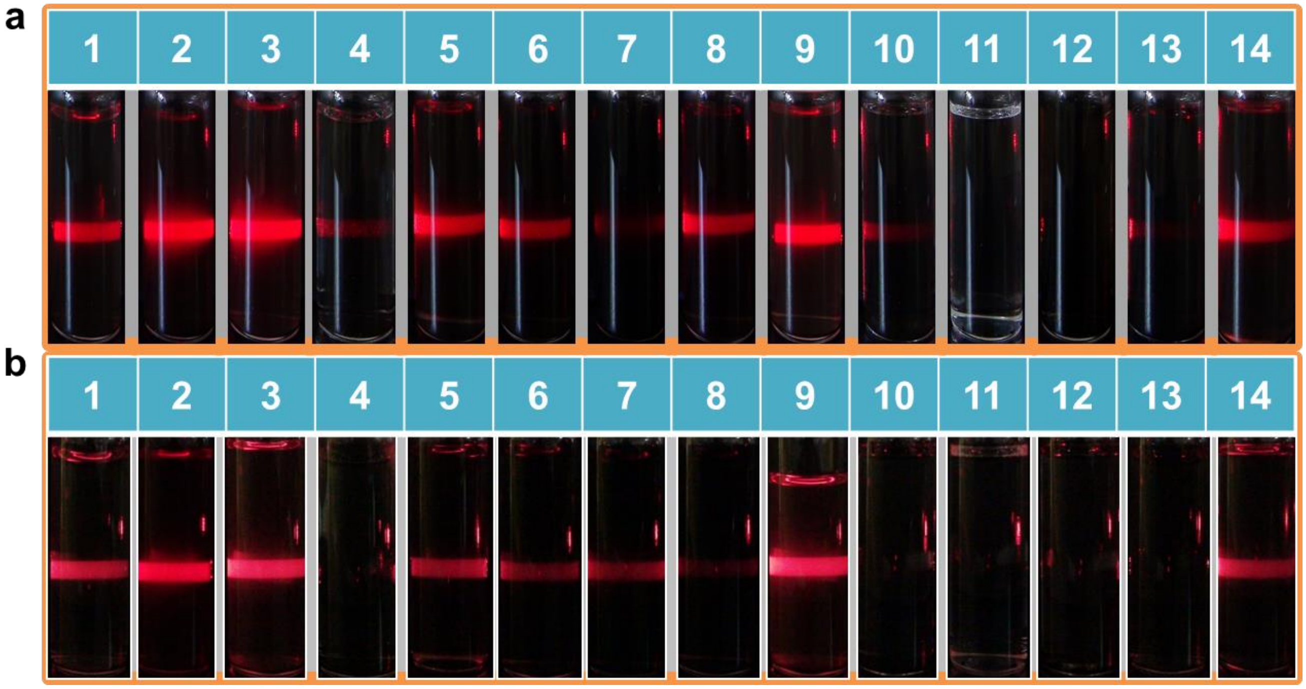

Supplementary Figure 3 | Solubility of the C_2N-*h*2D crystal in various solvents: **(a)** After 30s. **(b)** After 1 week. (1) water; (2) acetic acid; (3) MeOH; (4) 1M HCl; (5) NMP; (6) DMF; (7) DMSO; (8) DMAc; (9) acetone; (10) toluene; (11) THF; (12) 1M NaOH; (13) benzene; (14) dichlorobenzene.

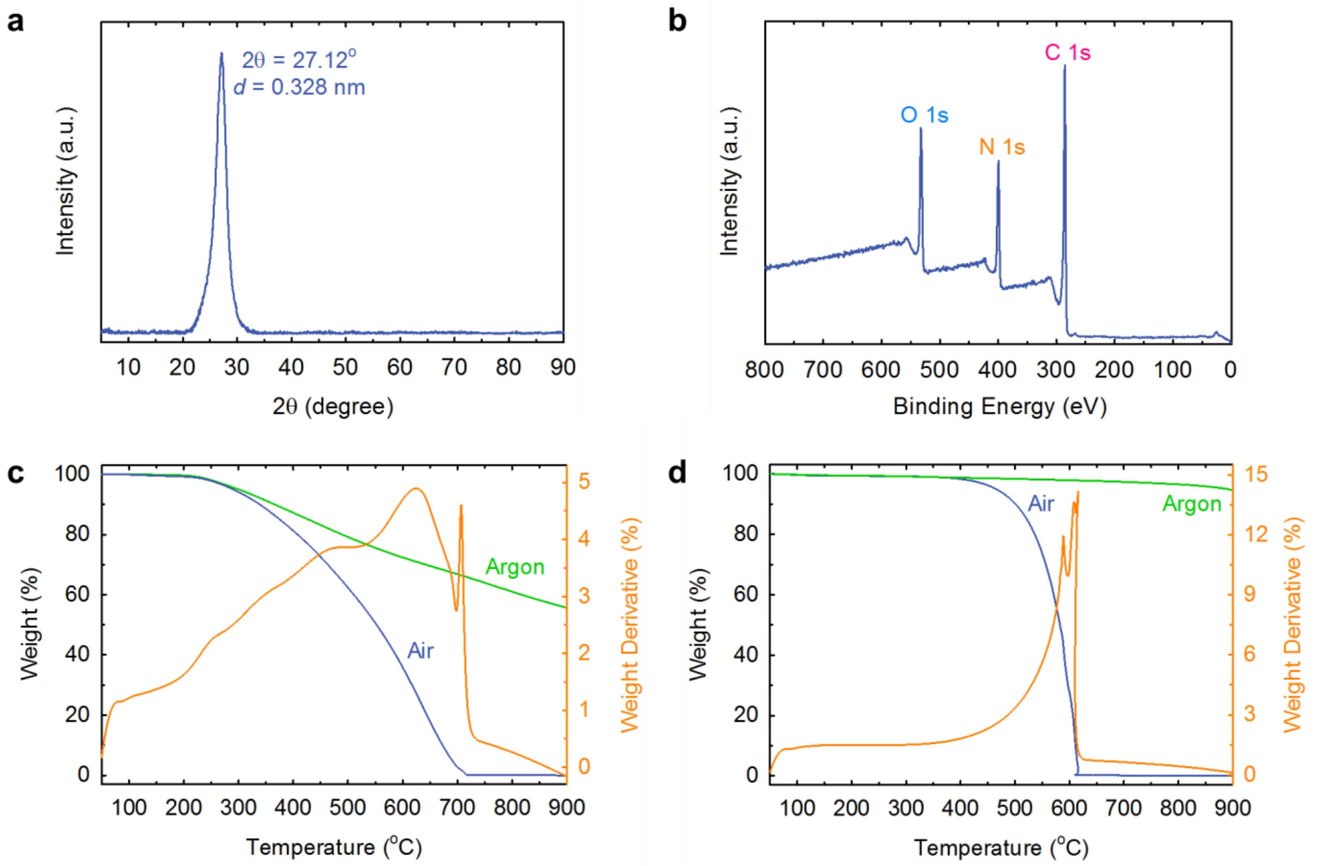

Supplementary Figure 4 | Analysis of the as-prepared C_2N-*h*2D crystal: **(a)** Powder XRD pattern from the as-prepared sample. **(b)** XPS survey spectrum showing C1s, N1s and O1s. High resolution XPS spectra for C1s, N1s and O1s are presented in Fig. S5. TGA thermograms obtained with a heating rate of 10 ℃/min in air and argon: **(c)** As-prepared sample. **(d)** Heat-treated sample at 700 ℃.

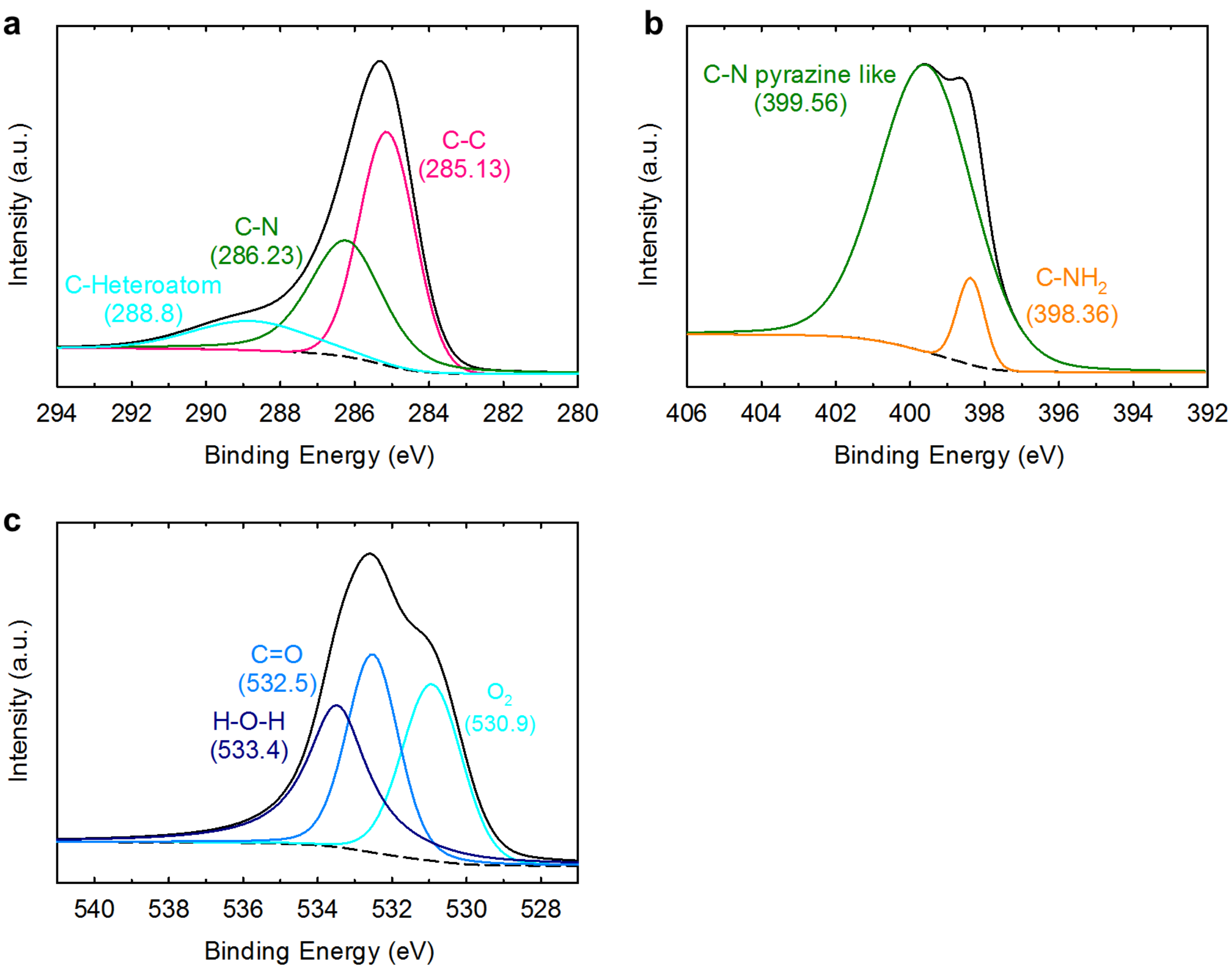

Supplementary Figure 5 | Deconvoluted XPS spectra of the C_2N-*h*2D crystal: **(a)** C 1s. **(b)** N 1s. **(c)** O 1s.

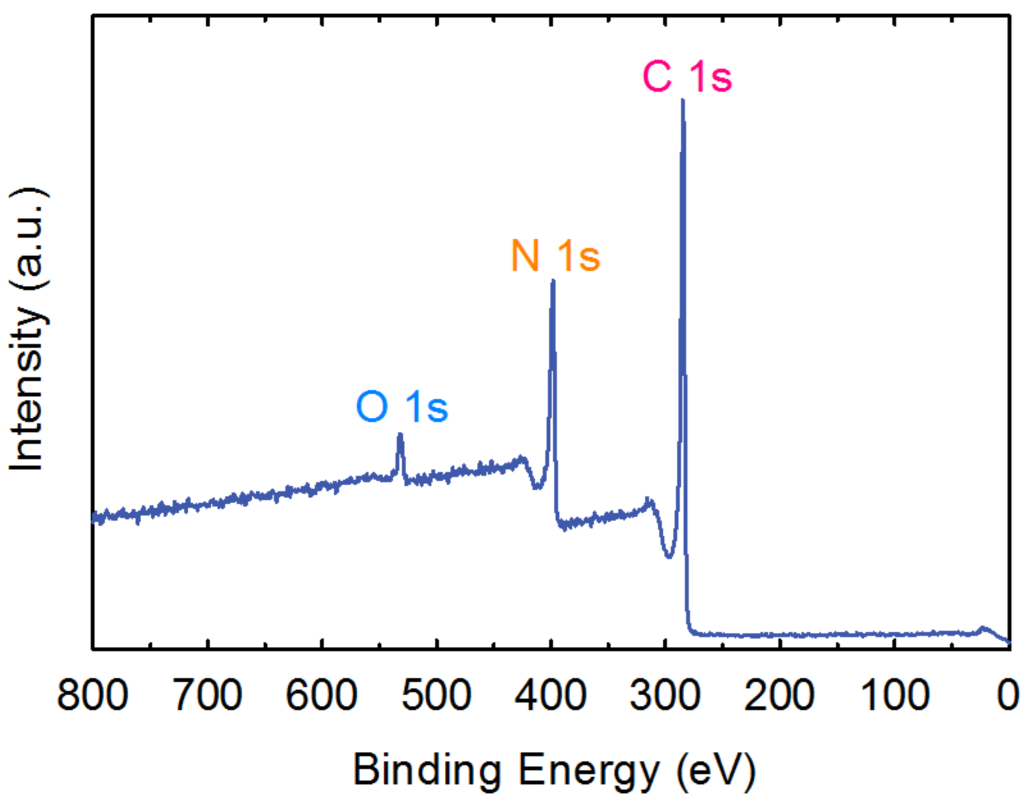

Supplementary Figure 6 | XPS survey spectrum of the heat-treated C_2N-*h*2D crystal at 700 ℃, showing C 1s, N 1s and reduced O1s compared with as-prepared spectrum (Fig. S4b).

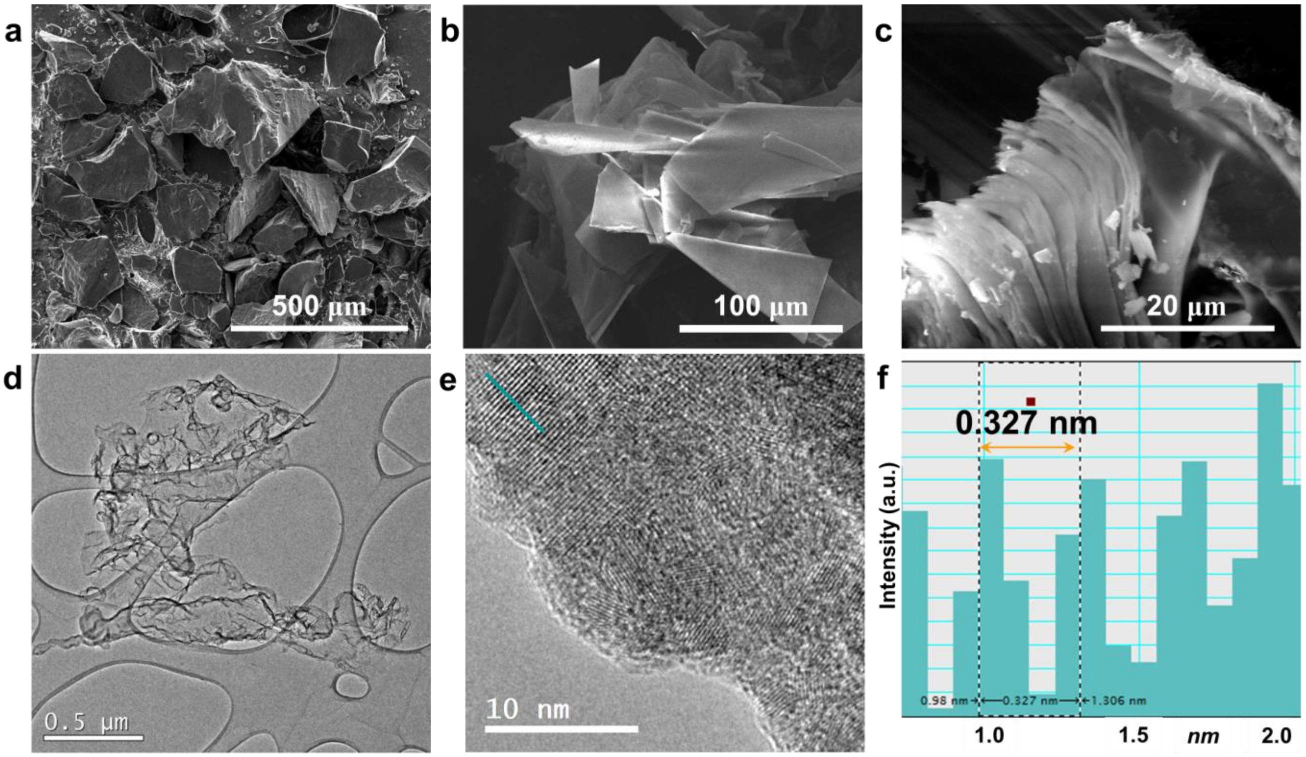

Supplementary Figure 7 | SEM images of the C_2N-*h*2D crystal: **(a)** As-prepared sample. **(b)** Heat-treated flakes. **(c)** Edge-on view of heat-treated flakes. TEM images of the C_2N-*h*2D crystal: **(d)** At low-magnification. **(e)** At high-magnification. **(f)** The interlayer distance (d = 0.327 nm) estimated along the dark green line in **(e)**. The interlayer distance of 0.327 nm is very close to that obtained from XRD pattern (0.328 nm, Fig. S4a).

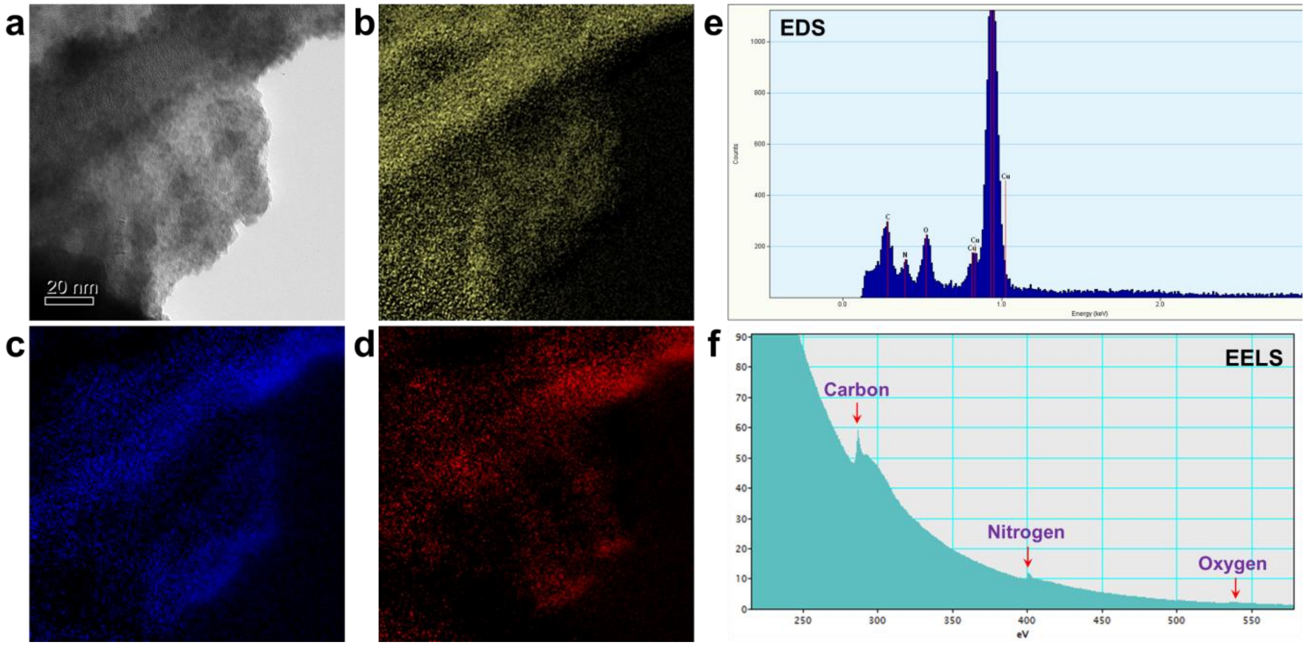

Supplementary Figure 8 | Energy-filtered TEM images of the as-prepared C_2N-*h*2D crystal: **(a)** Zero-loss image. **(b)** Carbon mapping. **(c)** Nitrogen mapping. **(d)** Oxygen mapping. **(e)** Energy dispersive X-ray spectroscopy (EDX) spectrum showing the elemental composition (C, N and O) from the TEM image **(a).** **(f)** Electron energy loss spectroscopy (EELS) spectrum from the TEM image **(a)**. Respective values of carbon, nitrogen and oxygen contents are very close to theoretical values.

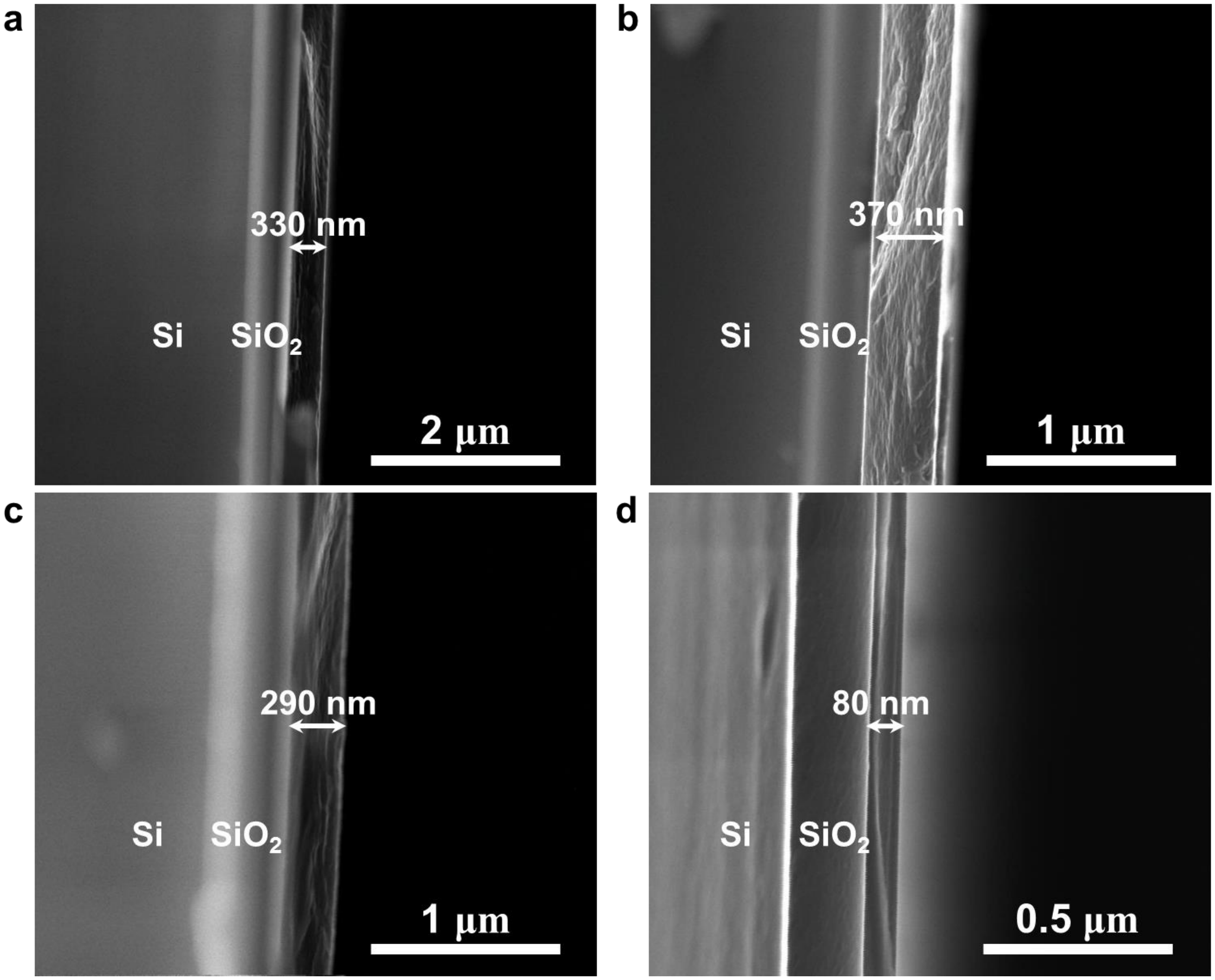

Supplementary Figure 9 | SEM images obtained from cross-section of the C_2N-*h*2D crystal films on SiO_2(300 nm)/Si wafer with different thickness: **(a)** 330. **(b)** 370. **(c)** 290. **(d)** 80 nm.

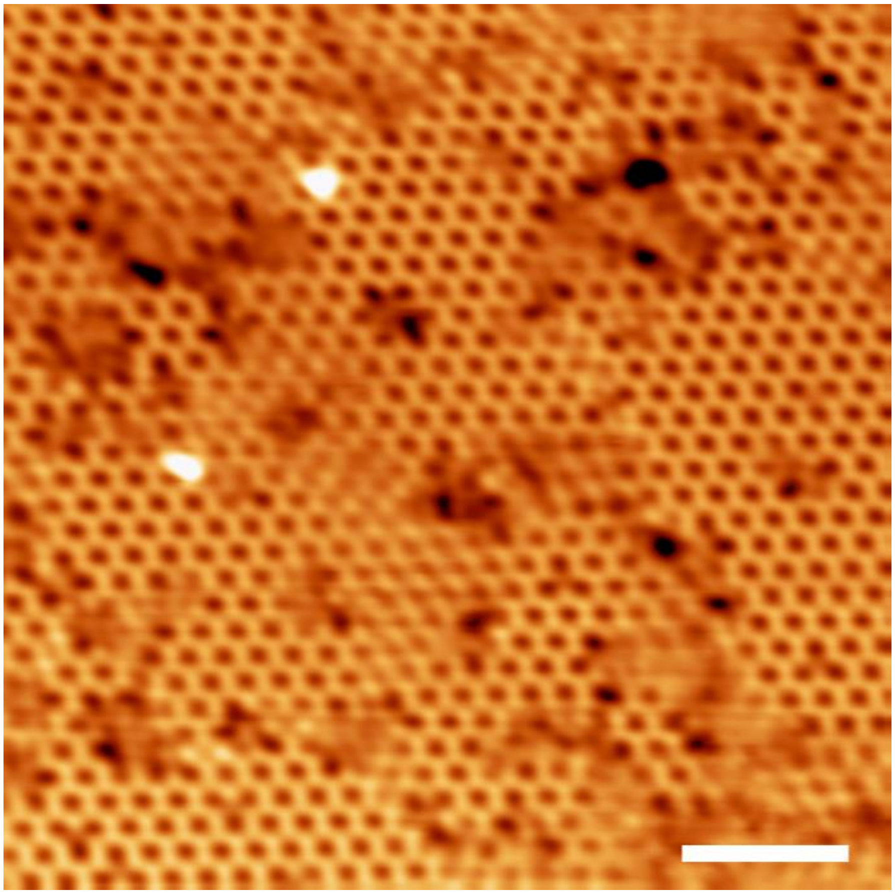

Supplementary Figure 10 | A large-area STM topography image of the C_2N-*h*2D crystal on Cu(111). High-resolution image is presented in Fig. 2a. Scale bar 5 nm.

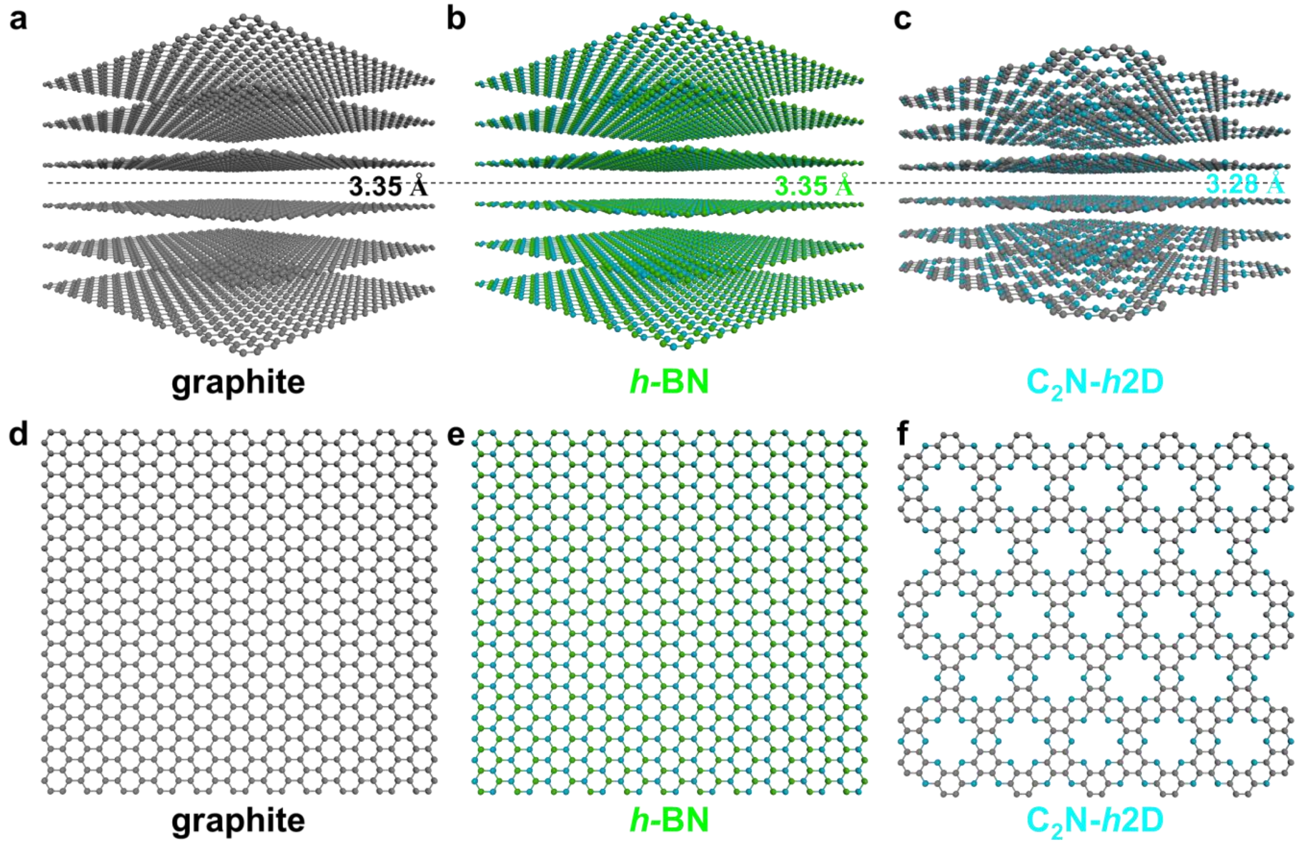

Supplementary Figure 11 | Comparison of interlayer distances: **(a)** Graphite. **(b)** *h*-BN. **(c)** C_2N-*h*2D crystal. Due to the differences in atomic sizes (covalent radii, B = 85, C = 77, N = 70 picometers), stacking patterns and charge polarizations, graphite and *h*-BN have very similar interlayer distance of 3.35 Å, while the C_2N-*h*2D crystal has lower interlayer distance (3.28 Å). Structures of representative single layer 2D crystals: **(d)** Graphene. **(e)** h-BN. **(f)** C_2N-*h*2D crystal.

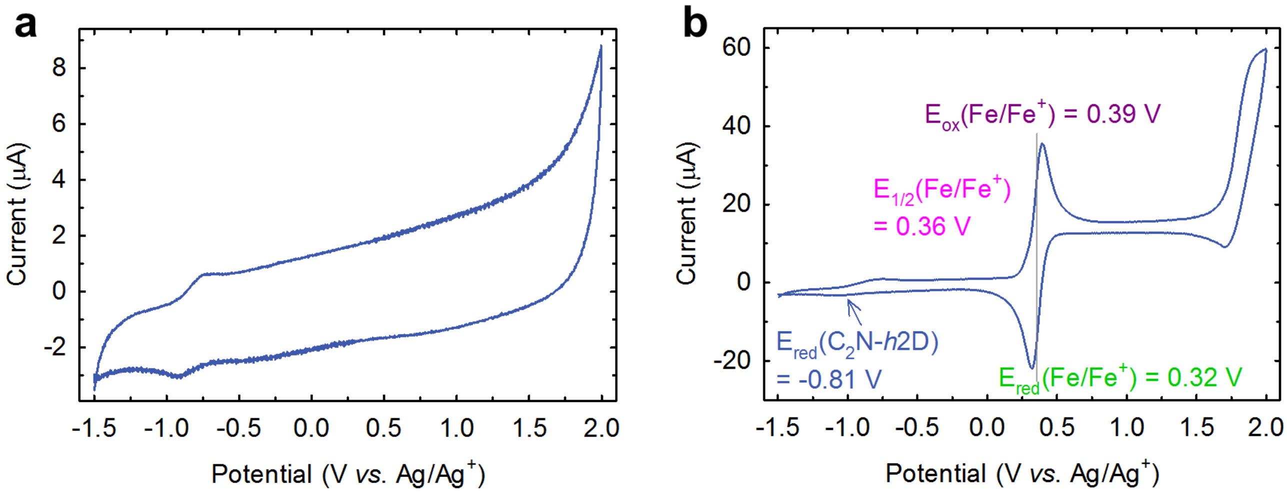

Supplementary Figure 12 | Cyclic voltammograms (CV) of the C_2N-*h*2D crystal on GC electrode at a scan rate of 100 mV s^{-1} using an Ag/Ag^+ reference electrode under N_2 atmosphere: **(a)** Whole range curve, showing no oxidation peak for the C_2N-*h*2D crystal. **(b)** C_2N-*h*2D crystal in the presence of ferrocene, which was used as reference material.

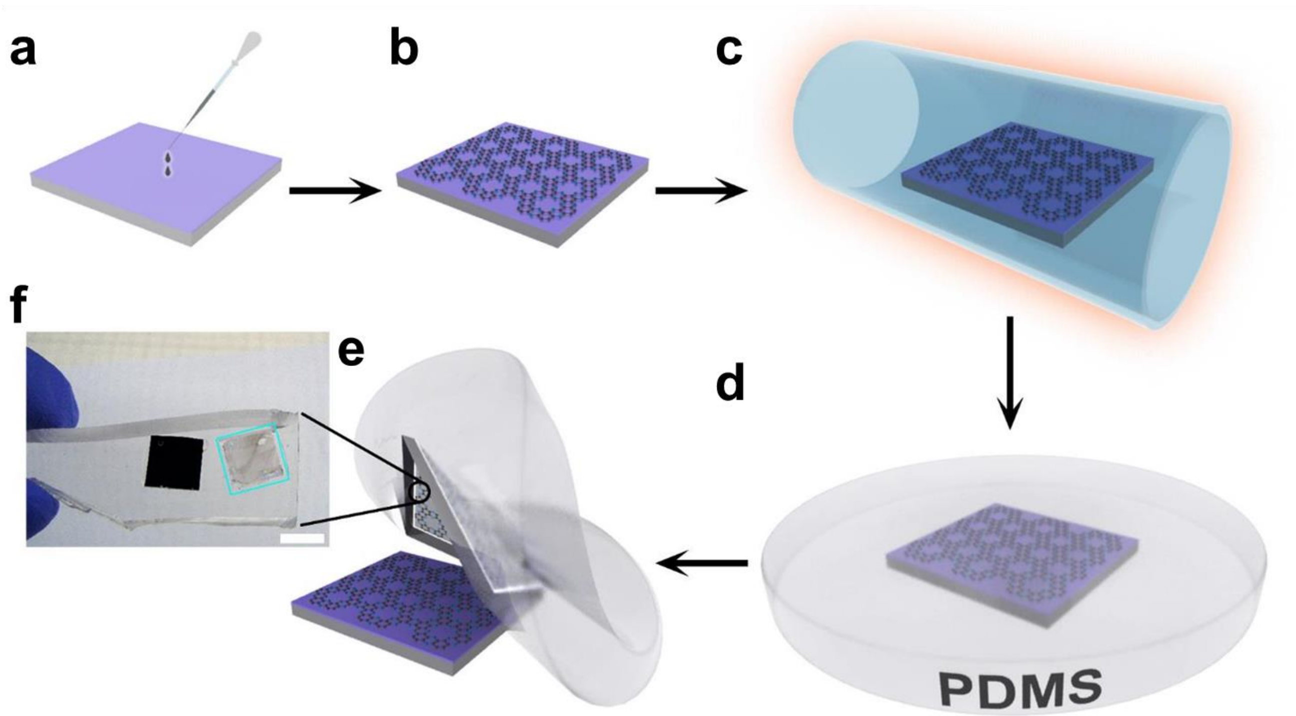

Supplementary Figure 13 | Schematic illustration for the film fabrication of the C_2N-*h*2D crystal using a PDMS mold: **(a)** 3–7 μL of the C_2N-*h*2D crystal solution was drop-coated onto the SiO_2/Si wafer using pipette. **(b)** The solution was slowly dried at 90 °C. **(c)** After cooling the sample to room temperature, the sample was annealed at 700 ℃ for 2 hours under argon atmosphere. **(d)** PDMS precursor was poured onto the sample film on SiO_2/Si wafer. **(e)** After curing the PDMS, which was carefully detached from the SiO_2/Si wafer. **(f)** Film attached to the PDMS after removing SiO_2/Si substrate is shown in the photograph (cyan blue square). Now the film can be transferred to any other desired substrate like SiO_2/Si wafer for device study. The scale bar is 1 cm.

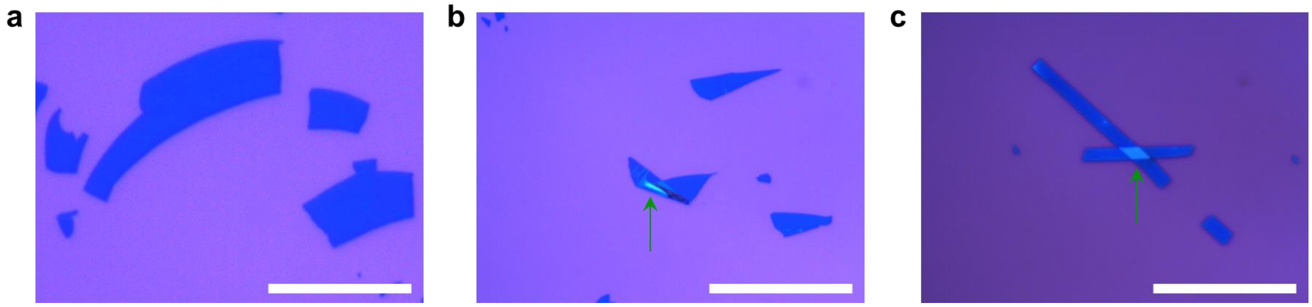

Supplementary Figure 14 | Optical images of the C_2N-*h*2D crystals on a SiO_2/Si wafer: **(a)** The C_2N-*h*2D crystal can be obtained over micron scale size. **(b)** The C_2N-*h*2D crystal is flexible (folded, cyan blue area). **(c)** Two C_2N-*h*2D crystal flakes have crossed (cyan blue area). All scale bars are 70 μm.

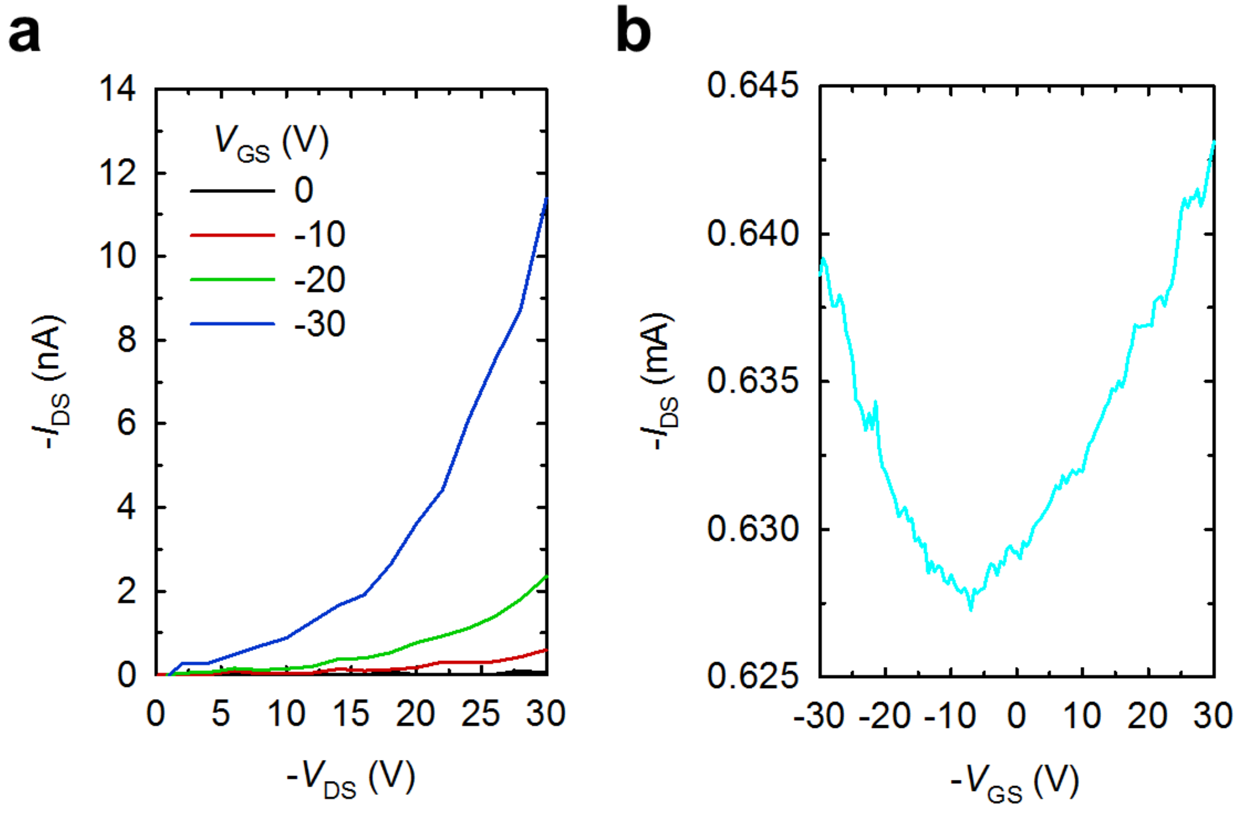

Supplementary Figure 15 | (a) Output curve after annealing at 100 °C under 5 × 10^{-6} torr (V_{DS} = -30 V). **(b)** Transfer curve of the C_2N-*h*2D crystal field-effect transistor before annealing. The characterization was performed at 25 °C under 5 × 10^{-6} torr.

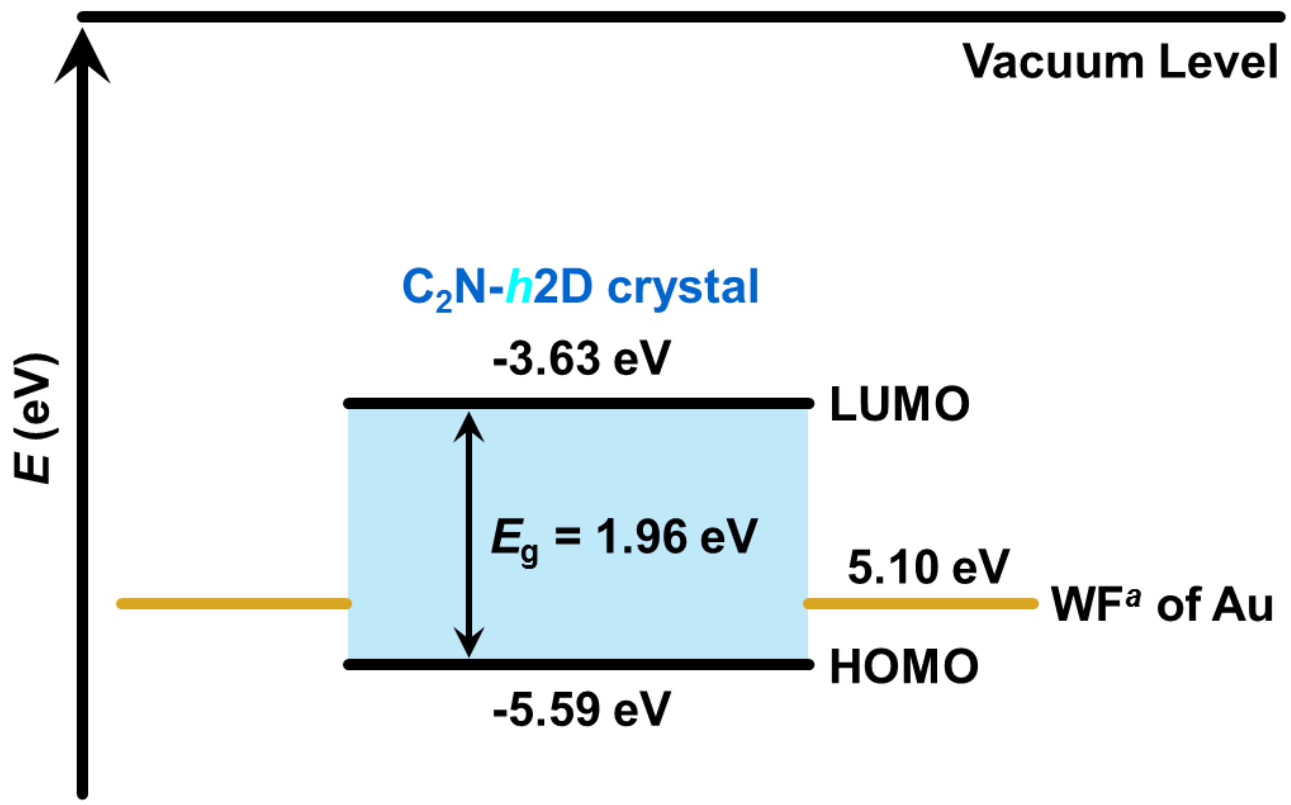

Supplementary Figure 16 | Energy level diagram of the C_2N-*h*2D crystal FET. The *p*-type operation of the FET is favorable with gold electrode according to the energy level diagram. [a] Work function.

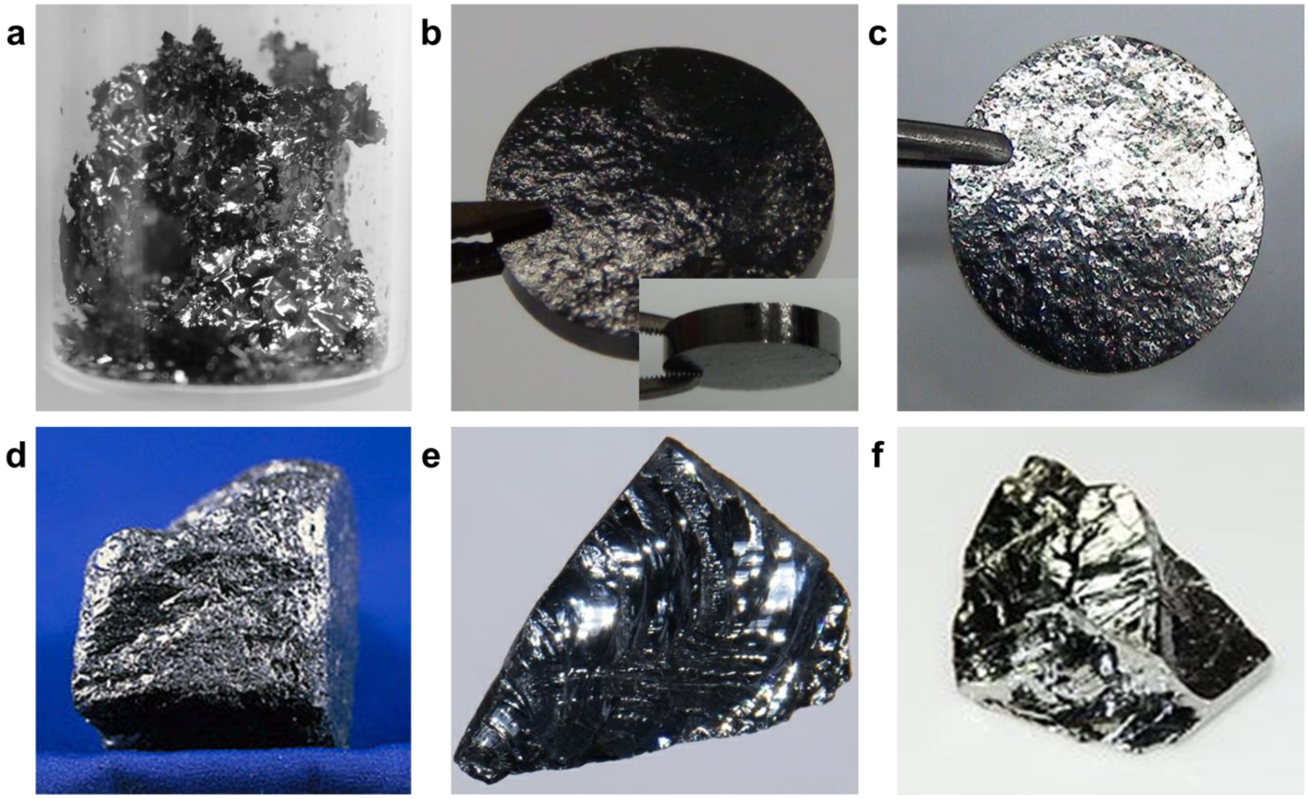

Supplementary Figure 17 | Photographs: **(a)** Heat-treated C_2N-*h*2D crystal at 700 ℃. **(b)** As-compressed C_2N-*h*2D crystal pellet by approximately 9000 bars. Inset is side-view of the pallet. **(c)** After annealing at 700 ℃. **(d)** Graphite ore (http://en.wikipedia.org/wiki/Graphite#cite_note-3). **(e)** Silicon ore (http://images-of-elements.com/silicon.php). **(f)** Germanium ore (http://images-of-elements.com/germanium.php). All cases are showing the surface in the shiny reflected light.

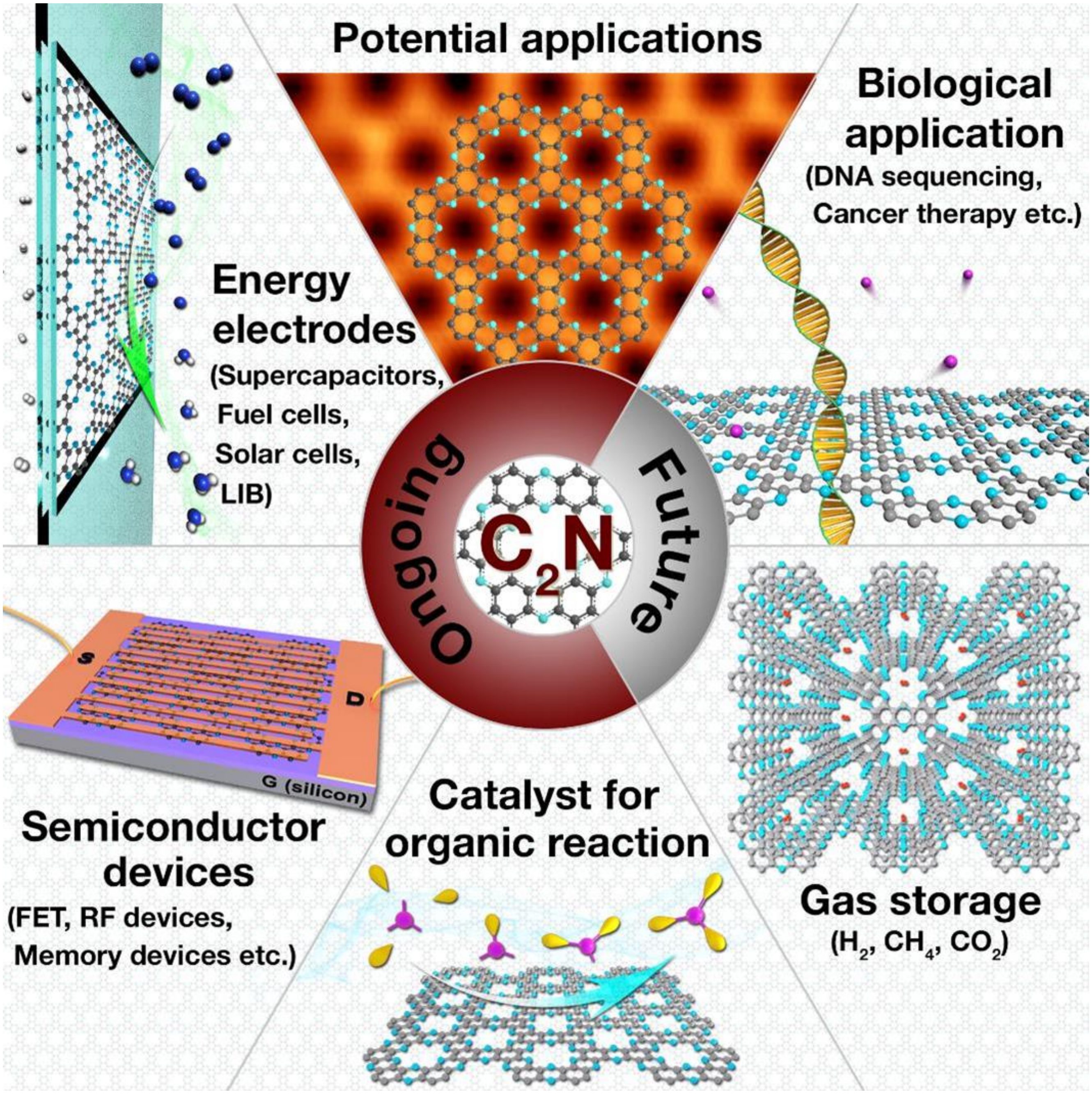

Supplementary Figure 18 | Potential applications of the C_2N-*h*2D crystal as a material for energy conversion and storage, semiconductor, catalyst, gas storage, biological and etc.

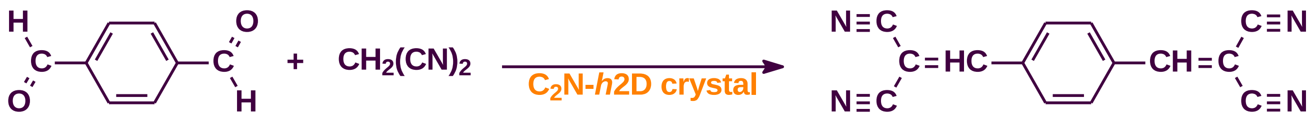

Supplementary Figure 19 | Schematic presentation of Knoevenagel condensation reaction of terephthaldehyde and malononitrile in the presence of the C_2N-*h*2D crystal as a reaction catalyst.

Supplementary Table 1 | Elemental composition of the C_2N-*h*2D crystal from different characterization techniques

**Technique**	**C**	**H**	**N**	**O**	**Total**
**Theoretical (wt%)**	54.54	1.54	31.81	12.11	100
**EA (wt%)**[a]	49.53	3.04	20.90	26.46	99.93
**XPS (at%)**[b]	66.49		17.89	15.62	100
**SEM EDS (wt%)**	47.07		31.44	21.49	100
**TEM EDS (wt%)**	52.93		35.96	11.11	100

[a] EA is most reliable element counts for bulk sample. The lower carbon content and the higher oxygen and hydrogen contents than theoretical calculation should be due to trapped small molecules (moisture, oxygen, and etc.) in the holes and interlayers of the C_2N-*h*2D crystal.

[b] XPS is more sensitive to surface chemical composition.

Supplementary Table 2 | Average (mean) electrical properties of the C_2N-*h*2D crystal field-effect transistors

**Electrical Characteristics**[a]	**Electrical Performance**
**Average off current**[b] **(A)**	7.1 (± 4.1)[c] × 10^{-14}
**Maximum on/off ratio**[d]	4.6 × 10^{7}
**Average on/off ratio**[e]	2.1 (± 3.9) × 10^{5}
**Maximum mobility ($cm^2V^{-1}s^{-1}$)**	1.3 × 10^{-2}
**Average mobility ($cm^2V^{-1}s^{-1}$)**	6.5 (± 0.2) × 10^{-3}

[a] 50 FET devices were measured under 5 × 10^{-6} torr.

[b] The off current was averaged over the turn-off state.

[c] The standard deviation.

[d] Calculated with the minimum off current.

[e] Calculated with the average off current.

Supplementary Table 3 | Comparison of material properties

Properties	C_2N-*h*2D crystal	Si	*h*-BN	Graphene/ graphite	Advantages for C_2N-*h*2D crystal	Ref.
**History**	2014	1824	1844	2004	New	14-17
**Band-gap (eV)**	1.96 (*c.a.*, 1.70)	1.12	5.05 - 6.40	0.00	Well in the range of Semi-conductor	18-22
**ON/OFF ratio**	$\sim10^7$	$\sim10^5$	--	<10	High ON/OFF ratio	23,24
**Dispersibility in solvents**	Good	--	--	Poor	Solution processable	25
**Bonds and structure**	C=C, C=N (*sp*^{2}) holey aromatic	Si-Si (*sp*^{3})	B-N	C=C (*sp*^{2}) aromatic	Heteroatom incorporated	
**Physical and electronic property**	Flexible semi-conductor	Brittle semi-conductor (SC)	Brittle insulator	Flexible conductor	2D semiconductor	
**Physical appearance**	Crystalline black powder	Shiny dark grayish solid	White solid	Crystalline black powder	--	
**Interlayer interactions**	π-π & dipole-dipole	--	--	π-π	Stronger interlayer interactions	26

Supplementary Note 1

In the high resolution C 1s spectrum, the dominant C 1s signals are centered at approximately 285.55 eV (graphitic sp^2C-C and sp^2C-N). The C1s spectra can be deconvoluted into 285.13, 286.23 and 288.8 eV (Fig. S5a). The peaks at 285.55 and 286.23 eV are assigned to sp^2C-C and sp^2C-N, respectively, in the aromatic ring, whereas the minor peak at 288.8 eV is attributed to the C-heteroatom, e.g., sp^2C=O, sp^2C-NH_2 at the edges (see structure **1** in Fig. S2). The N 1s spectrum shows only one major peak at 399.56 eV for the pyrazine-like nitrogen in the system and a very small peak at 398.36 eV for the C-NH_2 at the edges (Figs. S4b and S5b)[S1,2]. The presence of O 1s peak in the C_2N-*h*2D crystal can be attributed to the trapped substances such as moisture and O_2 in the holey structure (Fig. S5c), as well as the residual carbonyl (C=O) groups at the edges (see structure **1** in Fig. S2)[3]. Indeed, the heat-treated sample at 700 ℃ displayed a significantly reduced intensity of O 1s peak, while that of N 1s peak remained almost constant (Fig. S6). The result implies that the O1s peak before annealing has mostly contributed to the trapped substances in the holes and interlayers.

Supplementary Note 2

Measurement of LUMO and HOMO by cyclic voltammogram. To investigate the LUMO energy level of the C_2N-*h*2D crystal, the cyclic voltammogram was measured by using a standard three-electrode system, which consists of a glassy carbon as the working electrode, a platinum mesh as the counter electrode, and a silver wire as the reference electrode. Acetonitrile containing 0.1 M tetrabutylammonium hexafluorophosphate ($TBAPF_6$) was used as the supporting electrolyte. The LUMO energy level of the C_2N-*h*2D crystal was calculated according to the following equations (1-2):

Fc/Fc+: 0.36 V *v*s. Ag/Ag^+ (1)

LUMO (eV): $-4.8 - (E_{onset} - Fc/Fc^+) = -4.8 - (-0.81 – 0.36) = -3.63$ eV (2)

All electrochemical measurements were carried out under an ambient pressure and nitrogen atmosphere.

- Working electrode: glassy carbon
- Counter electrode: Pt mesh
- Reference: Ag wire
- Electrolyte: 0.1 M $TBAPF_6$ in acetonitrile
- Scan rate: 100 mV/s
- Preparation of the C_2N-*h*2D crystal on glassy carbon electrode: dropping and drying
- Fe/Fe^+: 0.36 *vs.* Ag/Ag^+
- E_{red} as -0.81 V,
- LUMO (eV) : -4.8 - (E_{onset} - Fc/Fc^+) = -4.8 - (-0.81 – 0.36) = **-3.63 eV**
- **HOMO (eV): -3.63 eV-1.96 eV* = -5.59 eV**

* The optical band-gap of -1.96 eV was used to estimate HOMO value.

The optical HOMO-LUMO gap was found to be 1.96 eV. The LUMO value can also be experimentally estimated by onset of the redox potentials taking the known reference level for ferrocene, 4.8 eV below the vacuum level according to the equation[4,5].

In our experiments, ferrocene exhibits an $E_{½}$ with a 0.36 V *vs.* Ag/Ag^+. The onset reduction potential of -0.81 V *vs.* Ag/Ag^+ was determined by electrochemical measurements of the C_2N-*h*2D crystal. Based on these data, the HOMO and LUMO can be calculated to be -5.59 and -3.63 eV, respectively. HOMO was calculated using the optical band-gap of 1.96 eV.

Supplementary Note 3

Fabrication of poly(dimethysiloxane) (PDMS) molds and FET device. An elastomeric PDMS molds were synthesized by curing PDMS precursor (Sylgard 184, Dow Corning Corp) on a heat

treated thin film of the C_2N-*h*2D crystal on SiO_2/Si substrate in a flat plastic petri dish. The PDMS precursor and curing agent (10:1 by weight) were mixed and degassed under vacuum to completely remove the bubbles. After curing overnight at 70 ℃ under vacuum, the film was transferred to the PDMS stamp from the SiO_2/Si substrate. The PDMS stamp is then positioned over the SiO_2/Si substrate and is pressed gently for about 20 second. Then the stamp is slowly removed so that the thinner flakes of C_2N will remain on the cleaned SiO_2/Si substrate for device fabrication. By PDMS stamping we were able to get very thin sheet of C_2N for device fabrication and transport studies. In brief, 3-7 μL of solution (concentration, 0.1 mg mL^{-1}) in trifluoromethanesulfonic acid (TFMSA) was drop casted onto a SiO_2/Si wafer. After the slow evaporation of the solvent at 140 °C, the SiO_2/Si wafer was annealed at 700 ℃. Then, a small amount of poly(dimethylsiloxane) (PDMS) precursor was poured onto the sample. After curing the PDMS, the SiO_2/Si wafer was detached away to obtain the sheets of the C_2N-*h*2D crystal on another SiO_2/Si wafer substrate on gently pressing. The resulting C_2N-*h*2D crystal flakes could be prepared over micron scale sizes (Fig. S14a) with mechanical flexibility (Fig. S14b). The crossing area of the stacked crystals was brighter than the other thin part (Fig. S14c). Gold electrodes of the transistors were patterned with conventional e-beam lithography (the typical channel width-to-length ratio (W/L) = 13, L = 500 nm) to construct bottom-gate top-contact FET. The hole motilities were extracted from the saturation regime of the transfer characteristics using the following equation (3):

$$I_{DS} = \frac{1}{2}\mu\frac{W}{L}C_i\,(V_{GS} - V_{th})^2 \qquad (3)$$

where I_{DS} is the drain current, μ is the field-effect mobility, C_i is the specific capacitance of the dielectric, V_{GS} is the gate voltage, V_{th} is the threshold voltage, and W and L are channel width and length.

Supplementary Methods

Materials. All the solvents, chemicals and reagents were purchased from Aldrich Chemical Inc., unless otherwise stated. Anhydrous NMP and glacial acetic acid were degassed with nitrogen purging before use. All the reactions were performed under nitrogen atmosphere using oven dried glassware. 2,4,6-Trinitroaniline[6], 1,3,5-triamino-2,4,6-trinitrobenzene[7] and 1,2,3,4,5,6-hexaaminobenzene trihydrochloride[8] were synthesized according to modified literature procedures.

C_2N-*h*2D crystal as a heterogeneous catalyst (Model reaction). The C_2N-*h*2D crystal efficiently catalyzes the Knoevenagel condensation reaction. Terephthaldehyde (2.00 g, 14.91 mmol) and malononitrile (1.97 g, 19.82 mmol) treated in benzene (50 mL) in the presence of the C_2N-*h*2D crystal (100 mg) as a reaction catalyst. The reaction mixture was kept on stirring at room temperature overnight under nitrogen atmosphere. Then the reaction mixture was filtered to simply remove the insoluble catalyst. The filtrate was dried by rotary evaporator to give a yellowish solid residue in quantitative yield. After recrystallized from ethanol, purified was 95%. The recovered C_2N-*h*2D crystal was repeatedly used three times, showing almost the same catalytic efficiency. ^{1}H NMR (600 MHz, DMSO) δ 8.08 (4H), 8.32 (2H); ^{13}C NMR (600 MHz, DMSO) δ 84.71, 112.75, 113.81, 130.85, 135.34, 159.81; HR-MS (ESI$^+$) Calcd. for ($C_{14}H_6N_4^+$) 230.0592, Found, 230.0591.

First-principle calculations. For computations, we used the Vienna *Ab initio* simulation package (VASP) to calculate the ground state of many electrons system in the frame work of density functional theory[9-12]. The plane-wave basis set with an energy cut-off of 400 eV and the PBE-type gradient-corrected exchange-correlation potential were employed[13]. The ions were described by the projector augmented wave (PAW) potentials. In the self-consistent-field total energy calculations, the k-points are uniformly sampled over the reciprocal space of the two-dimensional triangular lattice with mesh 21 by 21. All the atomic positions of were relaxed within residual forces smaller than 0.0eV/Å .

Supplementary References

1 Huang, H. G., Wang, Z. H. & Xu, G. Q. The selective formation of di-σ N−Si linkages in pyrazine binding on Si(111)-7 × 7. *J. Phy. Chem. B* **108**, 12560-12567 (2004).

2 Kou, Y., Xu, Y., Guo, Z. & Jiang, D. Supercapacitive energy storage and electric power supply using an aza-fused π-conjugated microporous framework. *Angew. Chem., Int. Ed* **50**, 8753-8757 (2011).

3 Yang, S., Feng, X., Wang, X. & Müllen, K. Graphene-based carbon nitride nanosheets as efficient metal-free electrocatalysts for oxygen reduction reactions. *Angew. Chem., Int. Ed* **50**, 5339-5343 (2011).

4 Wu, T.-Y., Sheu, R.-B. & Chen, Y. Synthesis and optically acid-sensory and electrochemical properties of novel polyoxadiazole derivatives. *Macromolecules* **37**, 725-733 (2004).

5 Kutner, W., Noworyta, K., Deviprasad, G. R. & D'Souza, F. Electrochemistry of solutions as well as simultaneous cyclic voltammetry and piezoelectric microgravimetry of conducting films of 2-(n-alkyl)fulleropyrrolidines. *J .Electrochem. Soc.* **147**, 2647-2652 (2000).

6 Hollernan, A. F. 1,3,4,5-Tetranitrobenzene. *Rec. trav. chim* **49**, 112 (1930).

7 Alexander R. Mitchell; Philip F. Pagoria; Robert D. Schmidt. Vicarious nuclecophilic substitution using 4-amino-1,2,4-triazole, hydroxyleamine or O-alkylhydroxylamine to prepare 1,3-diamino-2,4,6-trinitrobenzene or 1,3,5-triamino-2,4,6-trinitrobenzen synthesis and purification of 1,3,5-triamino-24,6-trinitrobenzene (TATB). USA 5633406 patent (1997).

8 Mahmood, J., Kim, D., Jeon, I.-Y., Lah, M. S. & Baek, J.-B. Scalable synthesis of pure and stable hexaaminobenzene trihydrochloride. *Synlett* **24**, 246-248 (2013).

9 Hohenberg, P. & Kohn, W. Inhomogeneous electron gas. *Phy. Rev.* **136**, B864-B871 (1964).

10 Kohn, W. & Sham, L. J. Self-consistent equations including exchange and correlation effects. *Phy. Rev.* **140**, A1133-A1138 (1965).

11 Kresse, G. & Furthmüller, J. Efficient iterative schemes for ab initio total-energy calculations using a plane-wave basis set. *Phy. Rev. B* **54**, 11169-11186 (1996).

12 Kresse, G. & Furthmüller, J. Efficiency of ab-initio total energy calculations for metals and semiconductors using a plane-wave basis set. *Comput. Mater. Sci.* **6**, 15-50 (1996).

13 Perdew, J. P., Burke, K. & Ernzerhof, M. Generalized gradient approximation made simple. *Phys Rev Lett* **77**, 3865-3868 (1996).

14 Emsley, J. *Nature's Building Blocks: An A-Z Guide to the Elements*. 387 (Oxford University Press, 2002).

15 Novoselov, K. S. *et al.* Electric field effect in atomically thin carbon films. *Science* **306**, 666-669 (2004).

16 Balmain, W. H., Remarks about the formation of compounds of boron and silicon with nitrogen and certain metals. *J. Prakt. Chem.* **27**, 422-430 (1842).

17 Haubner, R., Wilhelm, M., Weissenbacher, R. & Lux, B. Vol. 102 *Structure & Bonding* (ed Martin Jansen) 1-45 (Springer Berlin/Heidelberg, 2002).

18 Ribeiro, R. M. & Peres, N. M. R. Stability of boron nitride bilayers: Ground-state energies, interlayer distances, and tight-binding description. *Phy. Rev. B* **83**, 235312 (2011).

19 Bao, Q. & Loh, K. P. Graphene photonics, plasmonics, and broadband optoelectronic devices. *ACS Nano* **6**, 3677-3694 (2012).

20 Vel, L., Demazeau, G. & Etourneau, J. Cubic boron nitride: synthesis, physicochemical properties and applications. *Mater. Sci. Eng.: B* **10**, 149-164 (1991).

21 Robertson, J. Electronic structure and core exciton of hexagonal boron nitride. *Phy. Rev. B* **29**, 2131-2137 (1984).

22 Schwierz, F. Graphene transistors. *Nat. Nanotechnol.* **5**, 487-496 (2010).

23 Ahn, J.-H. *et al.* High-speed mechanically flexible single-crystal silicon thin-film transistors on plastic substrates. *Electron Device Lett., IEEE* **27**, 460-462 (2006).

24 Rafiq, M. A. *et al.* High on/off ratio and multimode transport in silicon nanochains field effect transistors. *Appl. Phys. Lett.* **100**, - (2012).

25 Englert, J. M. *et al.* Covalent bulk functionalization of graphene. *Nat. Chem.* **3**, 279-286 (2011).

26 Neto, A. H. C. & Novoselov, K. New directions in science and technology: two-dimensional crystals. *Rep. Prog. Phys.* **74**, 082501 (2011).
